# Efficient and metal catalyst-free approach towards highly-substituted *N*-sulfonylpiperidine-/mono-spiro-1,2,4,5-tetraoxanes for potential applications in chemotherapy

**DOI:** 10.1038/s41598-026-48982-6

**Published:** 2026-05-25

**Authors:** Mohit K. Tiwari, Malgorzata Kucinska, Agnieszka Zgoła-Grześkowiak, Philippe Grellier, Marek Murias, Łukasz Marczak, Lukasz Popenda, Tomasz Goslinski

**Affiliations:** 1 Chair and Department of Chemical Technology of Drugs, Poznan University of Medical Sciences, Rokietnicka 3, 60-806 Poznan, Poland; 2 Chair and Department of Toxicology, Poznan University of Medical Sciences, Rokietnicka 3, 60-806 Poznan, Poland; 3 Institute of Chemistry and Technical Electrochemistry, Poznan University of Technology, Berdychowo 4, 60-965 Poznan, Poland; 4 UMR7245 MCAM, CNRS, National Museum of Natural History, CP 52, 57 Rue Cuvier, 75005 Paris, France; 5 Institute of Bioorganic Chemistry, Polish Academy of Sciences, Noskowskiego 12/14, 61-704 Poznan, Poland; 6 NanoBioMedical Centre, Adam Mickiewicz University, Wszechnicy Piastowskiej 3, 61-614 Poznan, Poland

**Keywords:** Metal-free reaction, Tetraoxanes, Antiplasmodial activity, Cytotoxicity, Cancer, Chemical biology, Chemistry, Drug discovery

## Abstract

A highly efficient metal and catalyst-free approach for the preparation of synthetically challenged *tri*-OMe-aryl-substituted heterocyclic, *di*-/*tri*-OMe-aryl-based cyclic and acyclic 1,2,4,5-tetraoxanes (**5a–l** and **6a–k**) is presented herein. Under green-chemistry conditions, the reactions occur in two key steps. The first one involves azeotropic activation of hydrogen peroxide for *N*-sulfonylpiperidone (**2a–f**), substituted cyclic and acyclic ketones (**2a**_**1**_**–a**_**4**_) oxidation, whereas the second one was H^+^[BF_4_]ˉ (50–55% solun; 25 mol%)-catalyzed cyclization of the highly reactive *gem*-dihydroperoxide (**3a–f** and **3a**_**1**_**–a**_**4**_) intermediates in an SN_1_-type manner with secondary keto compounds (**4a–d**). Both reactions furnished a diverse array of heterocyclic, cyclic, and open-chain 1,2,4,5-tetraoxane analogues (**5a–l** and **6a–k**; 19–83%) in attainable yields. The outcomes of the control and competition experiment strongly indicate that the electronic effects of the EWGs group and the strong steric influence of the bulky *tri*-OMe-aryl group directly impacted reaction advancement. These effects were clearly observed in the reactivity of the substituted *N*-sulfonylpiperidones (**2a–f**) and *gem*-dihydroperoxides (**3a–f** and **3a**_**1**_**–a**_**4**_), and finally, also in the construction of the final products (**5a–l** and **6a–k**). In addition, this metal-free approach is also suitable for the ‘one pot’ synthesis of structurally complex, highly substituted, pharmaceutically privileged *di*-/*tri*-OMe-aryl-based cyclic and acyclic 1,2,4,5-tetraoxanes (**6a–h** and **6k**), offering significant potential for synthetic advancements in malaria and cancer chemotherapy. Furthermore, in preliminary biological evaluations, the in vitro antiplasmodial and cytotoxic potentials of novel *tri*-OMe-aryl-substituted *N*-sulfonylpiperidine-spiro-1,2,4,5-tetraoxanes (**5a–l**) were established against the chloroquine-resistant FcB1 strain of *Plasmodium falciparum* and human cancer cell lines, including HeLa and A2780 cells.

**Supplementary Information:**

The online version contains supplementary material available at 10.1038/s41598-026-48982-6.

## Introduction

**Fig. 1:**
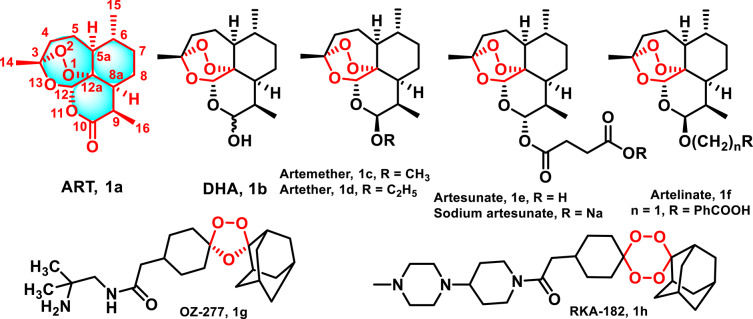
Representative examples of potent cyclic endoperoxides (**1a**–**h**).

Endoperoxides constitute a group of heterocycles that contain the characteristic peroxide bridges (O—O) and are prominent in numerous naturally derived secondary metabolites, including alkaloids, terpenoids and polyketides^1,2^. The crucial oxidizing feature of the endoperoxide bridges (by interacting with Fe(II)) is fundamental to the in situ generation of highly reactive free radical species and triggers interesting medicinal properties of the class, such as antitumor, antiparasitic (including antitrypanosomal and antischistosomal), as well as antiviral (SARS-CoV-2 COVID-19)^3–5^. In particular, the Chinese herb ‘*Artemisia annua*’ derived Artemisinin-based semisynthetic analogues are considered the Holy Grail of malaria chemotherapy, and in the form of Artemisinin-based Combination Therapy (ACT), used as the first line of defence against blood stage malaria (Fig. 1; **1a–f**)^6^. However, complex total synthesis procedures and the recent emergence of ART-resistant strains from Western Cambodia^7^ have driven the research into developing a fully synthetic version of the active pharmacophore^8^. In this context, three representative groups of synthetic endoperoxides, namely 1,2,4-trioxanes^9–11^, 1,2,4-trioxolanes^12^, and 1,2,4,5-tetraoxanes^13^, have been well-studied over the years and have been applied effectively for delivering novel antimalarial drug development candidates (Fig. 1; **1g–h**).

**Fig. 2:**
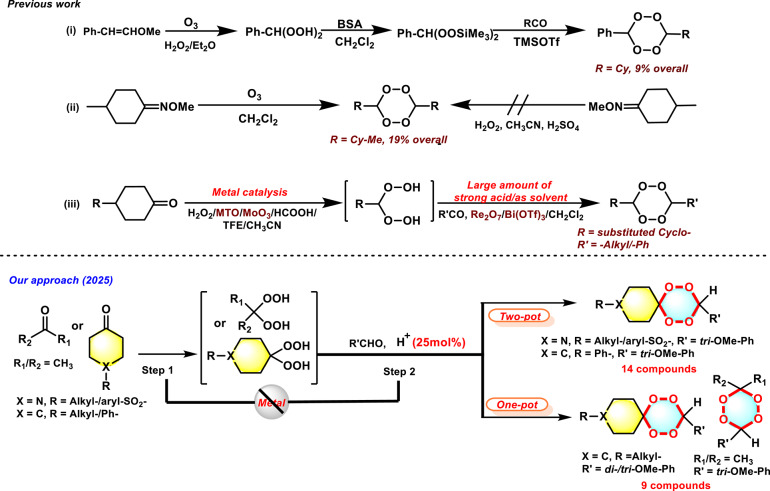
Classical and standarized approach for substituted mono-spiro-1,2,4,5-tetraoxanes. BSA-*N*,O-bis(trimethylsilyl)acetamide; MTO-methyltrioxorhenium(VII) complex; TFE-2,2,2-trifluoroethanol; TMSOTf-Trimethylsilyl trifluoromethanesulfonate.

Meanwhile, in the context of stability, efficacy, and oral bioavailability, recent studies have demonstrated the superiority of 1,2,4,5-tetraoxanes over other synthetic endoperoxides, including 1,2,4-trioxolanes^14^. Hence, various research groups have established diverse single (by ozonolysis of enolic ethers^15^, olefins^16^ and *O*-alkyl oximes^17^) or multistep (acid-catalysed cyclocondensation of explosive dihydroperoxide intermediate with carbonyl compounds^18^) routes to construct symmetric and non-symmetric 1,2,4,5-tetraoxane scaffolds endowed with two endoperoxide linkages (Fig. 2; scheme-i-ii). In literature, Rhenium (Re) salts, particularly MeReO_3_^19^ and Re_2_O_7_^20,21^, have been utilised as transition-metal catalysts for the direct synthesis of 1,2,4,5-tetraoxane. Since metal catalysis significantly improves the efficiency of the reaction, the challenges associated with the explosive dihydroperoxide intermediate separation were effectively overcome. In this regard, an efficient one-pot hydrogen peroxide (H_2_O_2_)/methyltrioxorhenium(VII) complex (MTO)/fluorous alcohol procedure for selective non-symmetric and potent dispiro-1,2,4,5-tetraoxanes synthesis was first reported by the Iskra group (Fig. 2; scheme-iii)^22^. The study revealed that a fluorous alcohol solvent activated both H_2_O_2_ and the methyltrioxorhenium catalyst, resulting in the exclusive formation of non-symmetric and dispiro-1,2,4,5-tetraoxanes. Notably, substituents at the 4-position of the carbonyl group were found to directly influence the reaction selectivity and the formation of competitive trimeric cyclic peroxides^23^. Later, P. M. O’Neill’s group applied this MTO-catalysed methodology and developed a wide range of potent dispiro-/*N*-sulfonylpiperidine-dispiro-1,2,4,5-tetraoxane analogues, including two antimalarial drug-development candidates, RKA-182 and E-209, with superior pharmacokinetic and pharmacodynamic properties and therapeutic activity (both in vitro and in vivo) against *P. falciparum* and *P. vivax* strains^24^. Parallelly, other efficient catalysts such as Bi(OTf)_3_,^25^ TMSOTf^26^, silica sulfuric acid^27^, and recently, MoO_3_^28^ have been screened for non-symmetrical 1,2,4,5-tetraoxane preparation (Fig. 2; scheme-iii)^29^. In some reports, strong acids such as sulfuric acid (H_2_SO_4_), perchloric acid (HClO_4_), tetrafluoroboric acid (HBF_4_), boron trifluoride (BF_3_) and their combinations have also been applied in stoichiometric concentration (as solvent media) to achieve the formation of symmetric and non-symmetric 1,2,4,5-tetraoxane moieties (Fig. 2; scheme-iii)^30,31^.

The prerequisite cautionary measures associated with handling expensive transition-metal catalysts, such as those that are air-, light-, and moisture-sensitive, as well as poorly functional-group-tolerant, often limit the utility of these reported protocols considerably. Furthermore, the hydroperoxide condensation step, which is carried out under hazardous, large-scale, strongly acidic conditions, also poses challenges. Besides, Iska’s group also explored I_2_-catalysed^32,33^ or azeotropic activation of aqueous hydrogen peroxide procedures^34^ for intermediate *gem*-dihydroperoxides preparation from simple cyclic and acyclic ketones. However, they have not further explored the advancement of the methodology in the synthesis of the privileged 1,2,4,5-tetraoxane scaffold. In addition, there are only a few reports related to the synthesis of potent substituted *N*-sulfonylpiperidine-spiro-1,2,4,5-tetraoxanes^35,36^. To the best of our knowledge, non-symmetric challenging *tri*-OMe-aryl-substituted *N*-sulfonylpiperidine-spiro-1,2,4,5-tetraoxane synthesis under complete metal-free conditions has not yet been reported.

Therefore, our observations have disclosed azeotropic activation of hydrogen peroxide as a powerful synthetic alternative for the preparation of highly reactive intermediates, i.e., *gem*-dihydroperoxides (**3a–f** and **3a**_**1**_**–a**_**4**_). Additionally, at a catalytic amount of tetrafluoroboric acid diethyl ether complex (HBF_4_.OEt_2,_ 50–55% solun; 25 mol%) in combination with 2,2,2-trifluoroethanol (TFE) solvent (fluorous alcohol) has proven efficient for the ring formation, yielding an array of challenging non-symmetrically *tri*-OMe-aryl-substituted heterocyclic, *di*-/*tri*-OMe-aryl-based cyclic and acyclic 1,2,4,5-tetraoxane analogues (**5a–l** and **6a–k**) under a greener metal-free environment (Fig. 2).

## Results and discussion

### Chemistry

We commenced our preliminary studies under a classical MTO-catalysed two-step one-pot manner^22^. Model substrates, i.e., 1-((4-(trifluoromethoxy)phenyl)sulfonyl)piperidin-4-one (**2d**, 0.3 mmol), H_2_O_2_ (35% solun; 1.5 mmol) and MTO (trace) in TFE solvent (4 mL), were allowed to react for 2 h at 25 °C. Then, the carbonyl compound 3,4,5-*tri*-OMe-aryl-aldehyde (**4d**, 0.45 mmol) and HBF_4_.OEt_2_ (50–55% solun; 0.3 mmol) were in situ added, and the reaction was further allowed to stir for an additional 1 h. After the specified time, neither the desired carbonyl hydroperoxidation nor in situ 1,2,4,5-tetraoxane ring formation was detected in thin-layer chromatography (TLC) (Table 1, entry 1).

**Table 1:** Optimization table: Screening of reaction parameters for substituted *N*-sulfonylpiperidone hydroperoxidation.^[a]^.


S. No	Solvent (mL)	Catalyst (mol%)	Acid (equiv.)	Oxidant (equiv.)	Temp (^o^C)	Time (h)	Conversion (%)^[b]^
1	TFE	MTO	HBF_4_.OEt_2_	H_2_O_2_	25	3	Nd
2	HFIP	MTO	HBF_4_.OEt_2_	H_2_O_2_	25	3	Nd
3	DCE	MTO	HBF_4_.OEt_2_	H_2_O_2_	25	3	Nd
4	DCM	I_2_	H_2_SO_4_	H_2_O_2_	25	3	Nd
5	ACN	MTO	HBF_4_.OEt_2_	H_2_O_2_	25	3	Nd
6	ACN	I_2_	–	H_2_O_2_	25	3	Nd
7.^[c]^	ACN	–	–	H_2_O_2_	25	3–6	15
**8.**^**[c]**^	**ACN**	–	–	**H**_**2**_**O**_**2**_	**25**	**18**	**96**
9.^[d]^	ACN	–	–	H_2_O_2_	25	18	46–81
10.^[e]^	ACN	–	–	H_2_O_2_	45	18	Trace

[a] *Reaction conditions*: **2d** (0.3 mmol), **4d** (0.45 mmol), catalyst (0.01–10 mol%), HBF_4_.OEt_2_ (50–55% solun; 0.3 mmol), and H_2_O_2_ (35% solun; 1.5 mmol) were reacted in solvent (4.0 mL) at 25 °C for 3 h. [b] The ^1^H NMR technique was used for % conversion calculation [% conversion = 100—(n_rs_/SM moles × 100)]; Where, n_rs_ = unreacted SM moles. [c] Step 1: **2d** (0.3 mmol) and H_2_O_2_ (35% solun; 1.5 mmol) were dissolved in two portions of acetonitrile (ACN:10 + 10 mL), subjected to azeotropic distillation (20 + 20 min) and left at 25 °C for 3 h. [d] H_2_O_2_ (2–4 equiv). [e] Mixture of products.

Next, other screened solvents, such as hexafluoro-2-propanol (HFIP), acetonitrile (ACN), 1,2-dichloroethane (DCE), dichloromethane (DCM) and MTO or I_2_ catalyst combinations were found to be inefficient in initiating the *N*-sulfonylpiperidone oxidation step (Table 1, entries 2–6). Subsequently, for the *N*-sulfonylpiperidone hydroperoxidation, we focused on azeotropic activation of the hydrogen peroxide approach^34^. Therefore, substituted *N*-sulfonylpiperidone (**2d**, 0.3 mmol) and H_2_O_2_ (35% solun; 1.5 mmol) were dissolved in two portions of acetonitrile (ACN; each 10 mL), concentrated under reduced pressure (20 + 20 min), and left at 25 °C for 3 h (Table 1, entry 7). Next, the traces of substituted *N*-sulfonylpiperidine-dihydroperoxide (**3d**) were identified at regular time intervals up to 6 h in TLC (Table 1, entry 7). The lower reactivity of *N*-sulfonylpiperidone (**2d**) towards oxidation prompted an extension of the reaction time from 6h to overnight. Thus, this time alteration significantly improved reaction efficiency, and the targeted *N*-sulfonylpiperidine-dihydroperoxide (**3d**) formation, with approximately complete consumption of the starting substrate (**2d**), confirmed by TLC (Table 1, entry 8). Notably, both reduction in the oxidant H_2_O_2_ (35% solun; 2–4 equiv) equivalents and the temperature variation had a detrimental impact on the oxidation process (Table 1, entries 9–10). Conceivably, numerous competitive side reactions promote the decomposition of reactive intermediate species crucial for hydroperoxidation, and the unreacted substrate (**2d**) remains persistent even after 18 h (Table 1, entries 9–10). On the other hand, a significant conversion was achieved with a slightly improved amount of the oxidant H_2_O_2_ (35% solun; 1.5 mmol) (Table 1, entry 8). The observance follows the established literature processes^34^.

Subsequently, the idea of one-pot condensation of *N*-sulfonylpiperidine-dihydroperoxide (**3d**) with secondary carbonyl compound (**4d**) in the presence of an acid (1 equiv) and fluorous alcohol did not succeed (Table 2, entry 1). The decomposition of reactive intermediates in the presence of 100% H_2_O_2_ is the most likely explanation for the failed condensation attempt. Hence, the remains of 100% H_2_O_2_ were first removed through routine workup, and then the purified crude reaction mass (**3d**), along with the second carbonyl derivative (**4d**), were again subjected to the same standard cyclisation conditions without any further chromatographic purification. After the specified time, the anticipated *tri*-OMe-aryl-substituted *N*-sulfonylpiperidine-spiro-1,2,4,5-tetraoxane (**5h**) scaffold was furnished with a particularly 63% significant yield (Table 2, entry 2). A similar equipotent pattern was also noticed with another fluorous alcohol-based solvent, HFIP, and the desired *tri*-OMe-aryl-substituted *N*-sulfonylpiperidine-spiro-1,2,4,5-tetraoxane (**5h**) was isolated in 59% yield (Table 2, entry 3). On further acid-catalyst loading experiments (10–50 mol%), unlike reported protocols^31,36^, we have identified that only a 25 mol% of HBH_4_.OEt_2_ solun in combination with fluorous alcohol (4 mL) is sufficient for effectively catalyzing the cyclization process and furnishes the desired *tri*-OMe-aryl-substituted *N*-sulfonylpiperidine-spiro-1,2,4,5-tetraoxane (**5h**) with an improved yield of 73% (Table 2, entry 4).

**Table 2:** Optimisation table: Screening of reaction parameters for substituted *N*-sulfonylpiperidine-dihydroperoxide cyclisation.^[a]^.


S.No	Solvent (mL)	Catalyst (mol%)	Acid (mol%)	Temp (^o^C)	Time (h)	Yield (%)^[b]^
1.^[c]^	TFE	–	HBF_4_.OEt_2_	25	1	Nd
2	TFE	–	HBF_4_.OEt_2_	25	1	63
3	HFIP	–	HBF_4_.OEt_2_	25	1	59
**4.**^**[d]**^	**TFE**	–	**HBF**_**4**_**.OEt**_**2**_	**25**	**1**	**73**
5	DCM	–	HBF_4_.OEt_2_	25	1	Nd
6	EtOH	–	HBF_4_.OEt_2_	25	1	Nd
7	H_2_O	–	HBF_4_.OEt_2_	25	1	Nd
8	ACN	–	HCl	25	1	Trace
9	EtOH	–	H_2_SO_4_	25	1	Nd
10	TFE	I_2_	–	25	1	Nd

[a] *Reaction conditions*: Step 2: **3d** (purified crude mass), **4d** (0.45 mmol), catalyst (10 mol%), HBF_4_.OEt_2_ (50–55% solun; 0.3 mmol) and solvent (4.0 mL) were allowed to react at 25 °C for 1 h. [b] Isolated yield. [c] **3d** (crude mass). [d] HBF_4_.OEt_2_ (50–55% solun; 25 mol%; 0.075 mmol).

The mmol of reactants and isolated yield of final products have been calculated as per the mmol of starting substrates.

Other screened strong acid-based-/catalysts-, such as hydrochloric acid (HCl), H_2_SO_4_, and or I_2,_ as well as solvents like H_2_O, ACN, DCM, and EtOH, were found to be ineffective in impacting the catalytic cycle (Table 2, entries 5–10). In continuation, variation in the gram equivalents of the secondary carbonyl (**4d**, 1–2 equiv) was also not beneficial and diminished the desired **5h** overall yield. Thus, azeotropic activation (ACN; 10 + 10 mL) of H_2_O_2_ (35% solun; 1.5 mmol) at 25 °C for 18 h, followed by HBF_4_.OEt_2_ (50–55% solun; 25 mol%; 0.075 mmol) catalysed cyclisation with the secondary carbonyl (**4d**, 0.45 mmol) in TFE (4 mL) at 25 °C for 1 h were recognised as the best-optimised transition metal-free greener conditions for substituted *N*-sulfonylpiperidone (**2d**) hydroperoxidation and *tri*-OMe-aryl-substituted *N*-sulfonylpiperidine-spiro-1,2,4,5-tetraoxane (**5 h**) preparation, respectively.

**Scheme 1:**
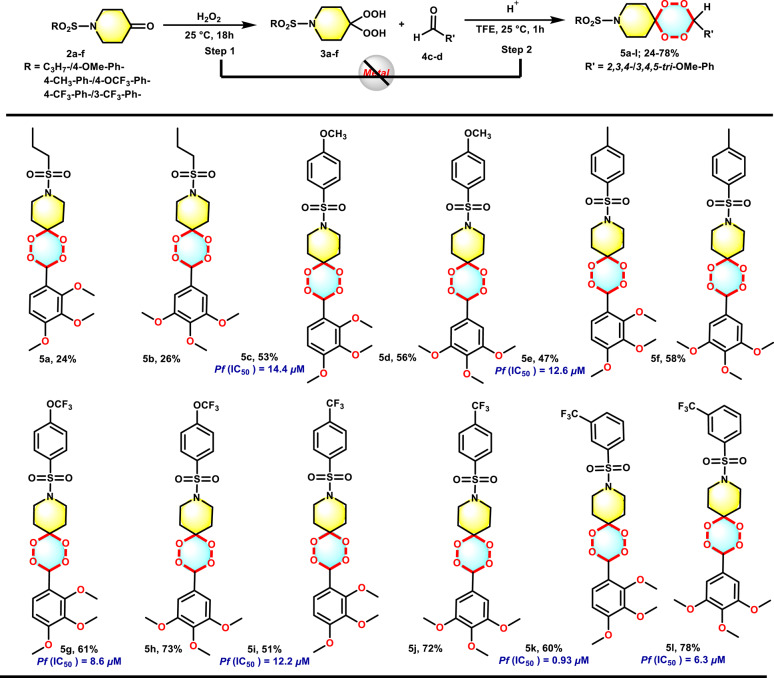
Synthesis of *tri*-OMe-aryl substituted *N*-sulfonylpiperidine-spiro-1,2,4,5-tetraoxanes **(5a–l)**. [a] *Reaction conditions*: **Step 1**: **2a–f** (0.3 mmol) and H_2_O_2_ (35% solun; 1.5 mmol) were dissolved in two portions of acetonitrile (ACN: 10 + 10 mL), subjected to azeotropic distillation (20 + 20 min) and left at 25 °C for 18 h; **Step 2**: **3a–f** (purified crude mass), **4c–d** (0.45 mmol), HBF_4_.OEt_2_ (50–55% solun; 25 mol%; 0.075 mmol) and TFE (4.0 mL) were allowed to react at 25 °C for 1 h. The initial antiplasmodial potential of the most potent analogues is highlighted in blue. IC_50_ value is the mean ± the standard deviation of at least three (n = 3) independent experiments.

Following this, the substrate scope exploration of the methodology was initiated with a set of alkyl-/aryl-substituted *N*-sulfonylpiperidones (**2a–f**)*.* Notably, the required alkyl-/aryl-based *N*-sulfonylpiperidones (**2a–f**) were prepared from **2a’** via the established base-promoted biphasic reaction process^37^. Then, these *N*-sulfonylpiperidones (**2a–f**) were allowed to react with highly substituted (2,3,4-/3,4,5-*tri*-OMe-aryl-aldehydes (**4c–d**) under optimised conditions, and an array of *tri*-OMe-aryl substituted *N*-sulfonylpiperidine-spiro-1,2,4,5-tetraoxanes (**5a–l**) was prepared in a 24–78% yield range (Scheme 1). The introduction of the propyl group significantly affected the reactivity of the alkyl-*N*-sulfonylpiperidone moiety (**2a**), yielding a mild yield range of the final products (**5a–b**: 24–26%) (Scheme 1). Next, aryl-*N*-sulfonylpiperidones (**2b–f**) containing various electron-donating (EDGs: 4-OMe-/4-CH_3_-) or electron-withdrawing (EWGs: 4-OCF_3_/4-CF_3_/3-CF_3_) groups were efficiently tolerated in the optimised conditions, and a wide range of non-symmetric *tri*-OMe-aryl-substituted *N*-sulfonylpiperidine-spiro-1,2,4,5-tetraoxanes (**5c–l**) was delivered (Scheme 1).

Various EDGs: 4-OMe- and 4-CH_3_-aryl-group containing *N*-sulfonylpiperidones (**2b–c**) were smoothly transformed to a variety of *tri*-OMe-aryl-substituted *N*-sulfonylpiperidine-spiro-1,2,4,5-tetraoxanes (**5c–f**: 47–58%) under standardized conditions (Scheme 1). Notably, the substitution of different EWGs: 4-OCF_3_/4-CF_3_/3-CF_3_-aryl-group on *N*-sulfonylpiperidone skeleton (**2d–f**) proved to be advantageous for stabilizing the reaction intermediates, resulting in a significant upsurge in the yield of the final products (**5g–l**: 51–78%) (Scheme 1). It is noteworthy that the bulky 3,4,5-*tri*-OMe-aryl-aldehyde (**4d**) followed an interesting reactivity pattern and gave a higher yield of desired products (**5b, d, f, h, j, l**: 26–78%) than the corresponding 2,3,4-*tri*-OMe-aryl-based analogues (**5a, c, e, g, i, k**: 24–61%) (Scheme 1). Particularly, the reaction of more complex 3-CF_3_-aryl-*N*-sulfonylpiperidone (**2f.**) with 3,4,5-*tri*-OMe-aryl-aldehyde (**4d**) proceeded efficiently under standardized conditions, giving an excellent yield of 78% of the synthetically challenged 3,4,5-*tri*-OMe-aryl-substituted 3-CF_3_-aryl-*N*-sulfonylpiperidine-spiro-1,2,4,5-tetraoxane (**5l**) (Scheme 1).

**Scheme 2:**
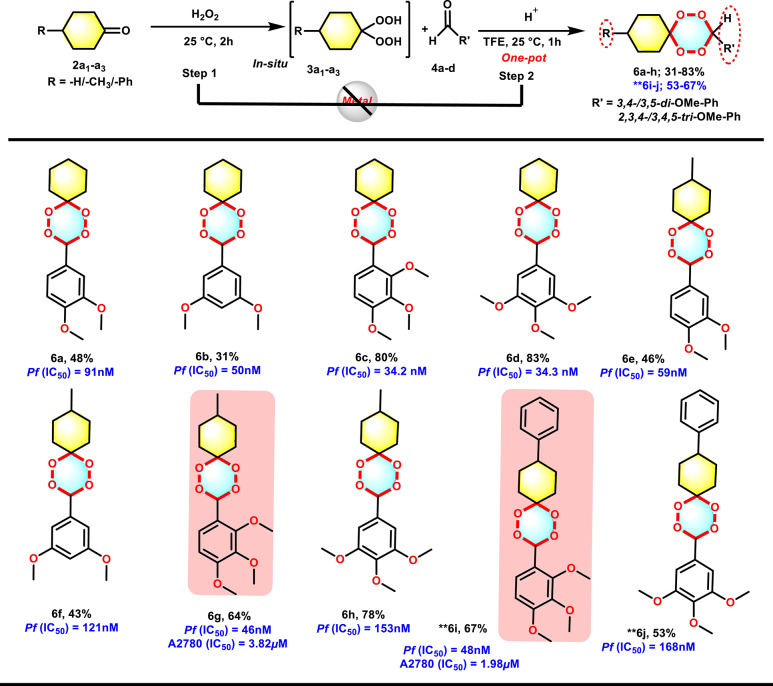
One-pot synthesis of C-4 substituted *di-/tri-*OMe-aryl-spiro-1,2,4,5-tetraoxanes **(6a-h)**. [a] *Reaction conditions*: **Step 1**: **2a**_**1**_**-a**_**2**_ (0.3 mmol) and H_2_O_2_ (35% solun; 1.5 mmol) were dissolved in two portions of acetonitrile (ACN: 10 + 10 mL), subjected to azeotropic distillation (20 + 20 min) and left at 25 °C for 2 h; **Step 2**: **4a–d** (0.45 mmol), HBF_4_.OEt_2_ (50–55% solun; 25 mol%; 0.075 mmol), and TFE (4.0 mL) were *in*-*situ* added and allowed to react at 25 °C for 1 h. **Both C-4 phenyl substituted compounds (**6i-j**; 53–67%) were prepared utilizing a two-step protocol as described in Scheme 1. The dual potent analogues (**6 g** and **6i**) from our recent studies are highlighted^38^. IC_50_ value is the mean ± the standard deviation of at least three (n = 3) independent experiments.

In a series of further investigations, the suitability of the optimised method was also investigated with simple substituted cyclic ketones (**2a**_**1**_**–a**_**3**_). Interestingly, both reactions, i.e., the cyclic ketones (**2a**_**1**_**–a**_**2**_) oxidation and the condensation of the in-situ formed highly reactive *gem*-dihydroperoxides (**3a**_**1**_**–a**_**2**_) with secondary substituted (3,4-/-3,5-)-*di-*/(2,3,4-/-3,4,5)*-tri*-OMe-aryl-aldehydes (**4a–d**), quickly proceeded in a one-pot sequence under optimised neutral transition-metal-free conditions (Scheme 2). Similar to our earlier observations^38^, the strong steric influence of the bulky *tri*-OMe-aryl group (**4c–d**) significantly affected the progress of the organic transformation, and the corresponding C-4 substituted *tri*-OMe-aryl-spiro-1,2,4,5-tetraoxanes (**6c–d**, **6g–h**: 64–83%) were obtained in good to excellent yield (Scheme 2). In contrast, only moderate yields of the desired *di*-OMe-aryl-spiro-1,2,4,5-tetraoxanes (**6a–b**, **6e–f**: 31–48%) were attained from **4a–b** (Scheme 2). In addition, to the best of our knowledge, this is the first example in which the construction of complex *di*-/*tri*-OMe-aryl-spiro-1,2,4,5-tetraoxane structures (**6a–h**) has been achieved by a direct transition-metal-free acid-catalyzed (HBF_4_.OEt_2_, 50–55% solun; 25 mol%) one-pot approach (Scheme 2). Apart from this, the chemical inertness and less reactivity of 4-phenylcyclohexan-1-one (**2a**_**3**_) also affected the reaction progress, and C-4-phenyl substituted *tri*-OMe-aryl-spiro-1,2,4,5-tetraoxanes (**6i–j**: 53–67%) were prepared in acceptable yield via a two-step protocol as described in Scheme 1. Where complete consumption of the starting substrates was achieved over additional reaction time (Scheme 2).

**Scheme 3:**
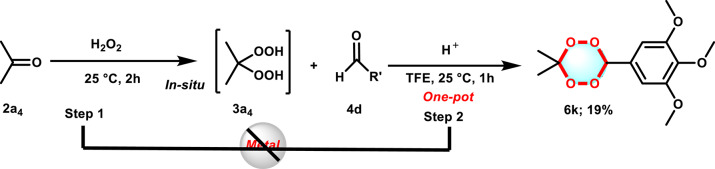
[a] *Reaction conditions*: **Step 1**: First, H_2_O_2_ (35% solun, 1.5 mmol) was dissolved in two portions of acetonitrile (ACN: 10 + 10 mL), subjected to azeotropic distillation (20 + 20 min). **Step 2**: Then, **2a**_**4**_ (0.3 mmol) was added to 100% H_2_O_2_ solun, and left at 25 °C for 2 h; **Step 3**: **4d** (0.45 mmol), HBF_4_.OEt_2_ (50–55% solun; 25 mol%; 0.075 mmol), and TFE (4.0 mL) were added *in*-*situ* and allowed to react at 25 °C for 1 h.

A one-pot acyclic dimethyl ketone (**2a**_**4**_) hydroperoxidation followed by ring closing reaction of a highly reactive intermediate (**3a**_**4**_) with 3,4,5-*tri*-OMe-aryl-aldehydes (**4d**) afforded the desired product (**6k**) in chemically acceptable yield. It is worth noting that most acyclic ketones are volatile. Therefore, acyclic dimethyl ketone (**2a**_**4**_) was directly added to a freshly prepared 100% H_2_O_2_ solun and then allowed to react under standardised metal-free conditions (Scheme 3).

**Scheme 4:**
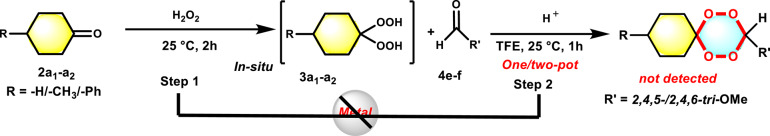
[a] *Reaction conditions*: **Step 1**: **2a**_**1**_**–a**_**2**_ (0.3 mmol) and H_2_O_2_ (35% solun, 1.5 mmol) were dissolved in two portions of acetonitrile (ACN: 10 + 10 mL), subjected to azeotropic distillation (20 + 20 min) and left at 25 °C for 2 h; **Step 2**: **4e–f** (0.45 mmol), HBF_4_.OEt_2_ (50–55% solun; 25 mol%; 0.075 mmol), and TFE (4.0 mL) were added *in*-*situ* and allowed to react at 25 °C for 1 h.

Surprisingly, the reaction of 2,4,5-*tri*-OMe-aryl-aldehyde (**4e**) and 2,4,6-*tri*-OMe-aryl-aldehyde (**4f.**) with ketones (**2a**_**1**_**–a**_**2**_) under optimised one-pot or two-pot conditions did not furnish the desired spiro-1,2,4,5-tetraoxane structures. The above observations confirmed that under defined conditions, a potential oxidation or side reactions initiate the self-degradation of highly reactive intermediates into different products (Scheme 4).

The compounds were characterised using mass spectrometry in the MS scan and MS/MS modes. Protonated, ammoniated, sodiated and doubly-charged molecules were found, and fragmentation analysis of synthesized compounds was performed. Purity was assessed using HPLC–UV analysis at wavelengths characteristic of each compound (see Supplementary Information). The results confirmed the successful synthesis of tetraoxanes (**5a–l and 6k**), which exist in achiral form, according to representative optical purity measurements performed using a digital refractometer for **5d** and **5h**.

**Scheme 5:**
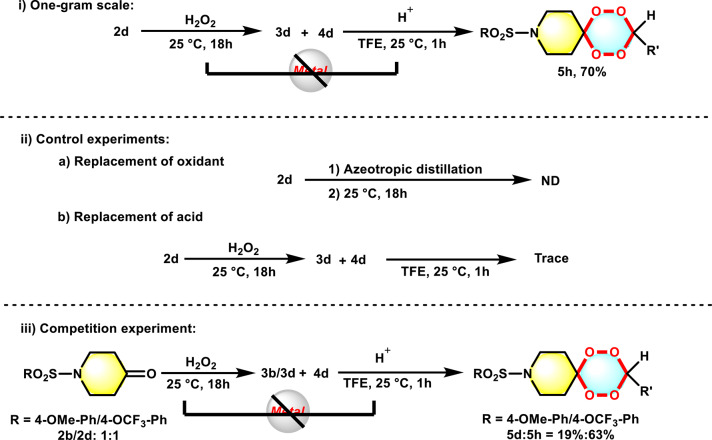
One-gram scale, control and competition experiments. [a] *Reaction conditions*: **Step 1**: **2b/2d** (0.3 mmol) and H_2_O_2_ (35% solun; 1.5 mmol) were dissolved in two portions of acetonitrile (ACN: 10 + 10 mL), subjected to azeotropic distillation (20 + 20 min) and left at 25 °C for 18 h; **Step 2**: **3b**/**3d** (purified crude mass), **4d** (0.45 mmol), HBF_4_.OEt_2_ (50–55% solun; 25 mol%; 0.075 mmol) and TFE (4.0 mL) were allowed to react at 25 °C for 1 h.

Next, the feasibility of the methodology was established on the gram scale, and an overall yield of 70% of 3,4,5-*tri*-OMe-aryl-substituted 4-OCF_3_-aryl-*N*-sulfonylpiperidine-spiro-1,2,4,5-tetraoxane (**5h**) was obtained (Scheme 5). Subsequently, various control experiments were conducted to assess the direct impact of the oxidant and acid-based catalyst on the key optimised hydroperoxidation and cyclisation steps. Since the absence of both H_2_O_2_ (5 equiv) and HBF_4_.OEt_2_ (50–55% solun; 25 mol%) completely aborted the organic transformation; the desired **5h** formation was not observed in the reaction mixture. In addition, the relative reactivity of different EDGs and EWGs (4-OCH_3_-aryl-/4-OCF_3_-aryl-) group-substituted *N*-sulfonylpiperidones toward the optimized conditions was determined in a one-pot competition experiment of **2b** and **2d**. Both the final 1,2,4,5-tetraoxanes **5d** and **5h** were isolated in 63% and 19% yield, respectively. The above one-pot competition experiment results revealed that the electronic effect of the EWGs group directly enhances the substituted *N*-sulfonylpiperidone (**2d**) reactivity and final product (**5h**) formation (Scheme 5).

Finally, an overall comparison of the optimised transition-metal-free approach with previously reported methods on various parameters is presented in Table 3.

**Table 3:** Significance of the transition-metal-free approach over the reported procedures.

Entry	Parameter	Optimized method	References
1	Sensitive metal catalyst loading	No	Yes^19–23^
2	Acid in catalytic quantity	Yes (25 mol%)	No^19–23,29–31^
3	Green solvent	Yes	Strong acids H_2_SO_4_, HCl, BF_3_ are also used^29–31^
4	Metal-free conditions	Yes	No^19–26^
5	Applicable for complex, Highly-substituted *N*-sulfonylpiperidine-spiro-1,2,4,5-tetraoxane synthesis	Yes	No^22,23,38^

**Fig. 3:**
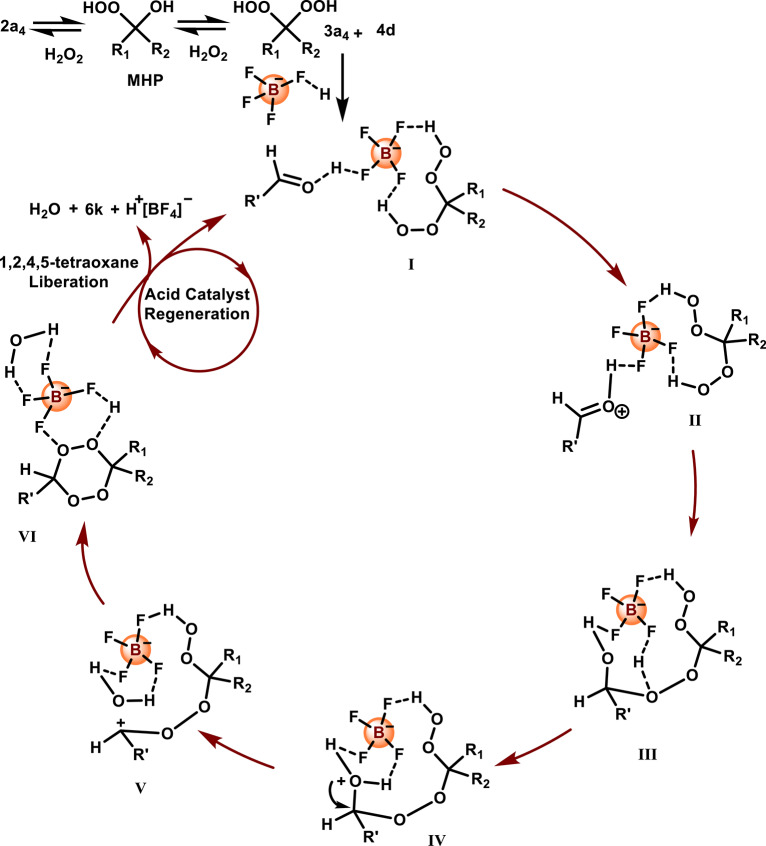
Plausible mechanistic pathway.

Based on preliminary experimental observations, a plausible mechanistic pathway for the ketone (**2a**_**4**_) oxidation and HBF_4_.OEt_2_ (50–55% solun; 25 mol%) catalysed reactive *gem*-dihydroperoxide (**3a**_**4**_) cyclisation is depicted in Fig. 3. Both steps were carried out in a transition-metal-free environment. First, azeotropic activation of the oxidant (H_2_O_2_ 35% solun) produced unstable perketal monohydroxy peroxide (MHP) from the ketone (**2a**_**4**_), which subsequently reacted with H_2_O_2_ to form highly reactive *gem*-dihydroperoxide (**3a**_**4**_)^34^.

In the second step, H^+^[BF_4_]ˉ interacts with *gem*-dihydroperoxide (**3a**_**4**_) and secondary aryl-ketone (**4d**) under neutral conditions to produce intermediate [I]. Subsequently, H^+^[BF_4_]ˉ-initiates protonation of the intermediate [I] and delivers intermediate [II]. The protonation of the carbonyl group of the secondary aryl-ketone (**4d**) substantially increases the reactivity of intermediate [II] to nucleophilic addition of hydroperoxide (**3a**_**4**_), leading to its transformation to intermediate [III]. Concurrently, H^+^[BF_4_]ˉ catalysed the extraction of the proton from [III] and the protonation of the hydroxyl group to yield intermediate [IV]. Following this, SN_1_-type water dissociation yields a tertiary carbocation intermediate [V]. Notably, available literature precedents have also suggested the formation of the carbocation intermediate^27^. Later, the second hydroperoxide of **3a**_**4**_ reacts with intermediate [V] to form [VI]. Finally, proton extraction from intermediate [VI] liberates the desired **6k** with H_2_O and regenerates the H^+^[BF_4_]ˉ to catalyse the next catalytic cycle.

### Antiplasmodial activity and cytotoxicity upon hela cells

#### In vitro antiplasmodial and cell cytotoxicity of non-symmetrical tri-OMe-aryl substituted N-sulfonylpiperidine-spiro-1,2,4,5-tetraoxanes (5**a–l**) against Pf FcB1 strain and HeLa cells

The preliminary in vitro antiplasmodial and cell cytotoxicity of *tri*-OMe-aryl substituted *N*-sulfonylpiperidine-spiro-1,2,4,5-tetraoxanes (**5a–l**) was determined against the chloroquine-resistant FcB1 strain of *Plasmodium falciparum* and HeLa cells, respectively. The results of the initial screening are presented in Table 4.

**Table 4:** In vitro antiplasmodial and cell cytotoxic activities of *tri*-OMe-aryl-substituted *N*-sulfonylpiperidine-spiro-1,2,4,5-tetraoxanes(**5a–l**) against *Pf* FcB1 and HeLa cells.

Code	Tetraoxanes	Antiplasmodial activity (*Pf* FcB1 strain IC_50_ (µM))		Cell cytotoxicity HeLa LD_50_ (µM)		Selectivity index (SI) (LD_50_ HeLa /IC_50_ *Pf* FcB1)
		IC_50_	SD	LD_50_	SD	
**5a**		> 25	–	> 50	–	> 2
**5b**		> 25	–	> 50	–	> 2
**5c**		14.4	0.7	> 50	–	3.5
**5d**		> 25	–	> 50	–	> 2
**5e**		12.6	1.4	50.9	9.1	4.0
**5f**		> 25	–	> 50	–	> 2
**5g**		8.6	1.0	27.3	4.5	3.2
**5h**		> 25	–	> 50	–	> 2
**5i**		12.2	1.0	17.0	5.1	1.4
**5j**		> 25	–	> 50	–	> 2
**5k**		0.927	0.15	> 100	–	107.88
**5l**		6.3	0.2	> 100	–	15.88
**1a**	**ART**	0.0056	0.0005	ND	ND	ND

*IC_50_: Concentration at which the inhibition of 50% cells was observed. **LD_50_: Median lethal dose. ***SI: Selectivity index: Ratio of LD_50_ against HeLa cells to the IC_50_ against *Pf* FcB1 strain. IC_50_ value is the mean ± the standard deviation of at least three (n = 3) independent experiments^39–41^.

In the initial study, *tri*-OMe-aryl-substituted propyl group-based *N*-sulfonylpiperidine-spiro-1,2,4,5-tetraoxanes (**5a–b**; *Pf* (IC_50_) =  > 25 µM) showed moderate antiplasmodial activity against the chloroquine-resistant FcB1strain of *Plasmodium falciparum*. Similarly, the EDGs: 4-OMe-/4-CH_3_-aryl group containing 3,4,5-*tri*-OMe-aryl substituted analogues (**5d** and **5f**; *Pf* (IC_50_) =  > 25 µM) showed lower potency. However, their corresponding 2,3,4-*tri*-OMe-aryl based analogues (**5c** and **5e**; *Pf* (IC_50_) = 12.6–14.4 µM) showed promising antiplasmodial activity in the micromolar range against the drug-resistant strain. A similar pattern of reactivity was also observed with EWG: 4-OCF_3_, 4-CF_3_-aryl group containing derivatives, where 2,3,4-*tri*-OMe-aryl substituted analogues (**5g** and **5i**; *Pf* (IC_50_) = 8.6–12.2 µM) showed higher micromolar antiplasmodial activity than 3,4,5-*tri*-OMe-aryl based moieties (**5h** and **5j**; *Pf* (IC_50_) =  > 25 µM). Surprisingly, both (2,3,4-/3,4,5-)-*tri*-OMe-aryl substituted 3-CF_3_-aryl-*N*-sulfonylpiperidine-spiro-1,2,4,5-tetraoxanes (**5k**–**l**; *Pf* (IC_50_) = 0.93–6.3 µM) showed good to excellent antiplasmodial activity. Particularly, the most potent 2,3,4-*tri-*OMe-aryl substituted 3-CF_3_-aryl-*N*-sulfonylpiperidine-spiro-1,2,4,5-tetraoxane (**5k**; *Pf* (IC_50_) = 0.927 µM) moiety displayed antiplasmodial potency in the submicromolar range. Overall, the idea of more lipophilic 3,4,5-*tri*-OMe-aryl group substitution on alkyl-/aryl-*N*-sulfonylpiperidine-spiro-1,2,4,5-tetraoxane skeleton was less efficacious. The corresponding analogues (**5b**, **5d**, **5f**, **5h**, **5j**, **5l**; *Pf* (IC_50_) = 6.3–25 µM) exhibited moderate antiplasmodial activity. In contrast, the 2,3,4-*tri*-OMe-aryl group substitution was beneficial and significantly improved the antiplasmodial potency of the analogues (**5a**, **5c**, **5e**, **5g**, **5i**, **5k**; *Pf* (IC_50_) = 0.927–25 µM) against the chloroquine-resistant FcB1strain of *Plasmodium falciparum*.

It is worth noting that all the *tri*-OMe-aryl-substituted *N*-sulfonyl-spiro-1,2,4,5-tetraoxanes (**5a**-**l**) showed lower efficacy than reference drugs such as artemisinin (*Pf* (IC_50_) = 5 nM), RKA-182 (*Pf* (IC_50_) = 1.1 nM), and E-209 (*Pf* (IC_50_) = 5.1 nM) against *Plasmodium falciparum*^13^. The outcomes of the current SAR analysis encouraged pursuing further exploration and key structural modifications around heterocyclic architecture, which will strengthen pharmacophore requirements for future advanced molecules, including fine-tuning physicochemical properties and druggability*.*

### Cytotoxic activity towards human ovarian cancer cells

**Fig. 4:**
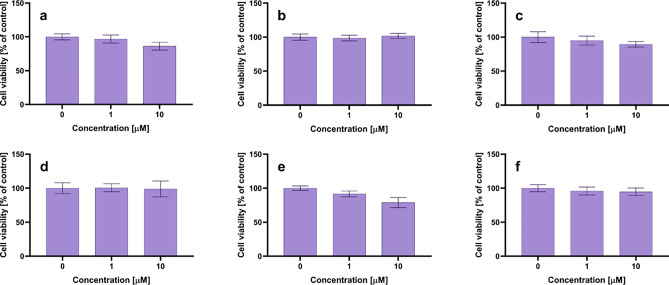
The cytotoxic activity of compounds **5a**–**5f**. The A2780 cells were treated with the tested compounds at concentrations of 1 μM and 10 μM for 72 h. The cell viability was measured using the MTT assay. The results are expressed as mean values ± SD from two (n = 2) experiments.

Previous studies have indicated that *N*-sulfonylpiperidinedispiro-1,2,4,5-tetraoxanes may exert strong antimalarial activity in both in vitro and in vivo models^35^. However, no studies have been conducted to evaluate the cytotoxic effects of these compounds on cancer cells. Thus, our study showed for the first time the activity of non-symmetrical *tri*-OMe-aryl-substituted heterocyclic 1,2,4,5-tetraoxanes (**5a**-**l**) towards human cancer cells. The compounds **5a**–**5f, 5j,** and **5k** did not significantly affect the cell viability in the preliminary studies. In contrast, compounds **5g**, **5i**, and **5l** reduced the cell viability to 66%, 65%, and 48% compared to control cells, respectively. The structure–activity relationship can show that the introduction of CF₃ or OCF₃ substituents at the *para* position in the *N*-sulfonylpiperidine moiety enhanced the cytotoxic effect compared to CH₃ or OCH₃ substituents; however, this effect was strongly dependent on the nature of the functional groups within the ring attached to the spiro-1,2,4,5-tetraoxanes scaffold. Substitution with OCH₃ groups at the *ortho*, *meta*, and *para* positions elicited a pronounced cytotoxic response, whereas substitution restricted to the *meta* (3,5) positions and *para* position completely abolished cytotoxic activity (compounds **5h** and **5j**). In contrast, when the CF_3_ moiety was placed in position *meta* of the *N*-sulfonylpiperidine moiety, only the derivative with methoxy groups introduced in positions meta (3,5) and *para* positions exerted the cytotoxic activity (Fig. 4).

**Fig. 5:**
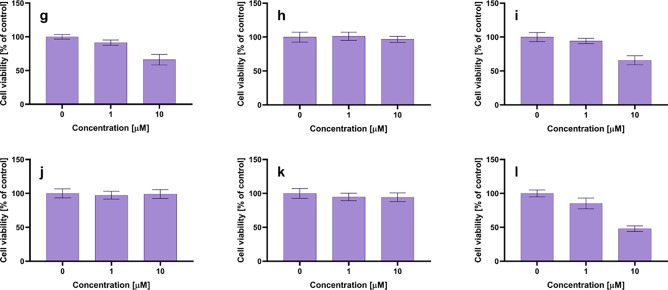
The cytotoxic activity of compounds **5g**–**5l**. The A2780 cells were treated with the tested compounds at concentrations of 1 μM and 10 μM for 72 h. The cell viability was measured using the MTT assay. The results are expressed as mean values ± SD from two (n = 2) experiments.

**Fig. 6:**
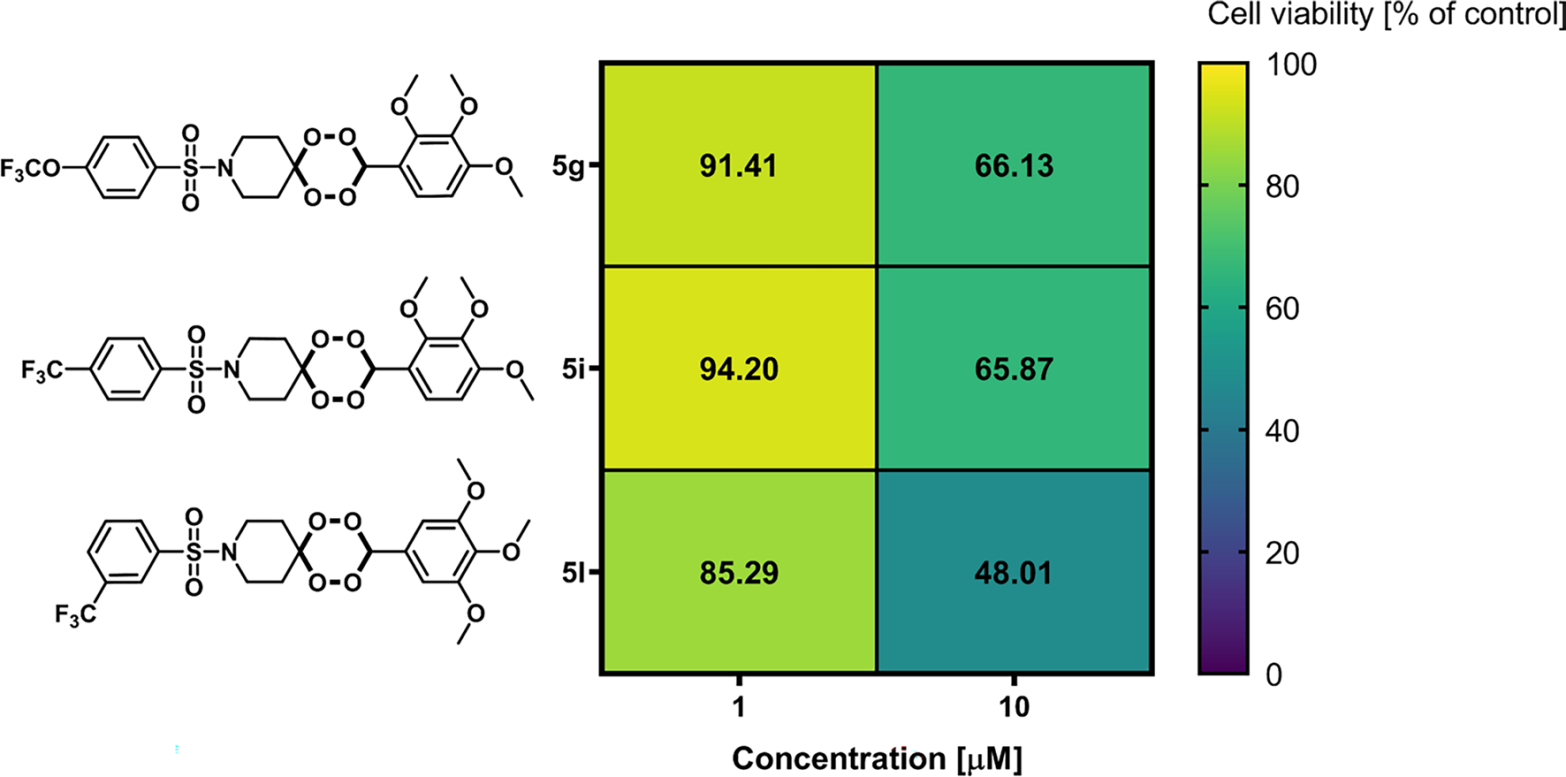
The cytotoxic effect of the most potent compounds towards A2780 cells. The results are presented as mean values from two (n = 2) experiments.

Among the twelve compounds (**5a-l**) tested, three (**5g**, **5i** and **5l**) reduced cell viability below 70% (Figs. 5 and 6). It should be emphasized, however, that compared to drugs commonly used for ovarian cancer treatment, these results suggest only moderate cytotoxic activity. Literature data indicate that the IC₅₀ of cisplatin for the A2780 cell line ranges between 0.65 and 9.55 μM^42–49^, whereas the most potent compound **5l** in this study reduced cell viability to approximately 50% only at the highest tested concentration (10 µM) (Fig. 6).

Therefore, it can be expected that its IC₅₀ would be around or above 10 μM. Thus, further modifications should focus on enhancing cytotoxic activity relative to the reference drug. Nevertheless, our study provides important new insights into the potential anticancer activity of *N*-sulfonylpiperidine-dispiro-1,2,4,5-tetraoxanes (**5a**–**l**) and highlights their potential as a promising scaffold for medicinal chemistry in anticancer research.

## Conclusion

In conclusion, we have documented an efficient, transition-metal-free protocol for challenging *tri*-OMe-aryl-substituted heterocyclic, *di*-/*tri*-OMe-aryl-based cyclic and acyclic 1,2,4,5-tetraoxanes (**5a–l** and **6a–k**) syntheses, which proceeds via carbonyl group oxidation and H^+^[BF_4_]ˉ(50–55% solun; 25 mol%)-catalyzed condensation with secondary ketones (**4a–d**). The structure–activity relationship (SAR) studies showed that the electronic effects of the EWGs: 4-OCF_3_/4-CF_3_/3-CF_3_-aryl-group on *N*-sulfonylpiperidone (**2d–f**) skeleton and the strong steric influence of the bulky *tri*-OMe-aryl group (**4c–d**) on *gem*-dihydroperoxide intermediates (**3a–f** and **3a**_**1**_**–3a**_**4**_) significantly affected the reactivity and proved beneficial to the progress of both organic transformations. In addition, the practicality of the methodology has been proven in terms of neutral conditions, functional group tolerance, control and competition experiments, gram-scale synthesis, and its applications in the synthesis of privileged *tri*-OMe-aryl-substituted heterocyclic, *di*-/*tri*-OMe-aryl-based cyclic and open-chain 1,2,4,5-tetraoxane (**5a–l** and **6a-k**; 19–83% yield range) scaffolds. The initial biological screening revealed a potent 2,3,4-*tri-*OMe-aryl substituted 3-CF_3_-aryl-*N*-sulfonylpiperidine-spiro-1,2,4,5-tetraoxane (**5k**; *Pf* (IC_50_) = 0.927 µM, SI = 107.88) analogue with submicromolar antiplasmodial potency and lower toxicity against the chloroquine-resistant FcB1 strain of *Plasmodium falciparum*. Furthermore, detailed studies on the mechanism of complex 1,2,4,5-tetraoxane (**5a–l** and **6a–k**) and other related medicinal chemistry aspects against various drug-resistant strains are currently ongoing in our laboratory and will be reported elsewhere soon.

## Experimental section

### General information

The glasswares were dried overnight in a laboratory hot air oven before its application in all synthetic procedures. All commercially procured AR-grade chemicals and solvents were used without additional purification and handled with specified safety measures (Commercial Source: Sigma Aldrich, Fluorochem, TCI). Both flash column chromatography and Analytical thin-layer chromatography were carried out on Merck silica gel (230–400 mesh size), Merck silica gel 60 F_254_ precoated plates (0.25 mm), respectively. For analysis, non-destructive TLC visualization techniques such as UV fluorescence and iodine vapor were used. For vacuum drying, the Heidolph Hei-VAP Core standard G1 rotavapor was used. The Stuart SMP10 laboratory melting point apparatus was utilized for melting point determinations and is presented uncorrected. The molecular structure and composition of the compounds were analyzed by NMR spectroscopy. The ^1^H (400 MHz) and ^13^C (100 MHz) NMR spectra were acquired on a Bruker Avance 400 spectrometer equipped with a 5 mm BBO probe, while the ^19^F (377 MHz) NMR spectra were recorded on an Agilent 400 MHz spectrometer equipped with a OneNMR probe. All spectra were measured at 25 °C in CDCl₃. Chemical shifts (δ) are reported in ppm with reference to TMS (δ = 0.00 ppm) for ^1^H and ^13^C NMR and to CFCl₃ (δ = 0.00 ppm) for ^19^F NMR. Coupling constants (*J*) are given in Hz, and multiplicities are abbreviated as follows: s (singlet), d (doublet), t (triplet), q (quartet) and m (multiplet).

Optical purity measurement experiments for compounds **5d** and **5h** were performed using a digital Atago RePo-1 refracto-polarimeter. The setup runs typically at ~ 3 mL sample size, and deionised water (DIW = 0.00) serves as the standard (baseline/blank) for each run. The optical rotation (α) measurements for **5d** and **5h** were performed in methanol.

The HPLC–DAD analyses were performed on the Summit HPLC system from Dionex, USA. For each compound chromatograms were collected at 254 nm and at two characteristic maxima to show whether a single chromatographic peak could be detected or the samples contained impurities. The peak purity was confirmed by comparing UV spectra at selected retention times under the peak. The analysis was performed on an Inertsil ODS-SP 250 × 4.6 mm, 5 µm column (GLSciences, Japan) that was thermostated at 35 °C. The following water–methanol gradient was performed: 0 min 80% methanol, 5 min 100% methanol, and 8 min 100% methanol. The flow rate was 1.5 ml/min and the injection volume was 5 µl. The low-resolution MS analyses, including compound fragmentation, were performed on the Q-TRAP 4000 mass spectrometer (AB-Sciex, USA) by direct injection of sample solutions to the ESI source utilizing a Harvard pump. The source was operated in the positive ionization mode. The working conditions were as follows: source temperature 450 °C, source voltage 4500 V, nebulizer gas–nitrogen at 40 psi, curtain gas–nitrogen at 10 psi, auxiliary gas–nitrogen at 45 psi, declustering potential 50 V, collision gas–nitrogen at 6 psi. For selected ions obtained from the MS scan spectrum, their fragmentations were recorded in the enhanced ion product mode.

High-resolution MS analysis was performed on a Bruker timsTOF Pro mass spectrometer (Bruker Daltonics, Bremen, Germany), equipped with a standard high-flow ESI ion source. The ESI source was operated at ± 4.5 kV, and nitrogen was used for nebulization (1.6 bar) and as a drying gas (8.0 l/min at 220 °C). External calibration was performed, enabling mass measurements with an accuracy of better than 5 ppm. For that purpose, a calibration mixture containing sodium formate clusters was used. The MS/MS spectra were collected at a frequency of 1 Hz for ions selected automatically based on the MS spectrum. The fragmentation energy depended on the molecular weight of the compounds studied and ranged from 10 to 25 eV. Data were collected using timsControl version 5.1.8. The data were analyzed using DataAnalysis version 6.1, provided by Bruker Daltonics.

### General procedures for preparation of alkyl-/aryl-substituted N-sulfonylpiperidone (**2a–f**): Preparation of 1-((4-(Trifluoromethoxy)phenyl)sulfonyl)piperidin-4-one (**2d**) as representative

A reaction mixture of 4-piperidone monohydrate hydrochloride (**2a’**, 500 mg, 3.26 mmol), 4-(trifluoromethoxy)benzenesulfonyl chloride (1.28 g, 4.89 mmol), K_2_CO_3_ (900 mg, 6.51 mmol) and CHCl_3_:H_2_O (1:1) (10 mL) was charged in a 50 mL RBF and allowed to react in a closed environment at rt for 18 h. After TLC confirmation, the reaction mixture was quenched using NaHCO_3_ (10 mL). Afterwards, the aqueous phase was extracted using dichloromethane (3 × 10 mL) and washed with brine (2 × 10 mL) solution and dried over anhydrous MgSO₄. Next, the removal of excess solvent from the organic phase was carried out in vacuo. Then, direct crystallization from the crude reaction mixture was initiated with a nonpolar solvent such as *n*-pentane or *n-*hexane under a cold atmosphere. Finally, pure white solid 1-((4-(trifluoromethoxy)phenyl)sulfonyl)piperidin-4-one (**2d**) was attained.

#### 1-(Propylsulfonyl)piperidin-4-one (**2a**)^35^

The crude product was purified by a direct crystallisation process using* n*-pentane or *n*-hexane as the crystallisation solvent. *R*_*f*_ value: 0.50, Yield: 87% (580 mg); White solid; m.p. 59–61 °C; ^1^H NMR (400 MHz, CDCl_3_) δ 3.63 (t, *J* = 6.1 Hz, 4H),  3.02–2.96 (m, 2H), 2.57 (t, *J* = 6.1 Hz, 4H), 1.92–1.82 (m, 2H), 1.08 (t, *J* = 7.5 Hz, 3H); ^13^C NMR (100 MHz, CDCl_3_) δ 205.95, 52.93, 45.58, 41.70, 17.06, 13.04.

#### 1-((4-Methoxyphenyl)sulfonyl)piperidin-4-one (**2b**)^35^

The crude product was purified by a direct crystallisation process using* n*-pentane or *n*-hexane as the crystallisation solvent. *R*_*f*_ value: 0.50, Yield: 91% (800 mg); White solid; m.p. 123–125 °C; ^1^H NMR (400 MHz, CDCl_3_) δ 7.76–7.71 (m, 2H), 7.03–6.99 (m, 2H), 3.88 (s, 3H), 3.38 (t, *J* = 6.2 Hz, 4H), 2.54 (t, *J* = 6.2 Hz, 4H),; ^13^C NMR (100 MHz, CDCl_3_) δ 205.70, 163.33, 129.68, 127.83, 114.49, 55.69, 45.89, 40.62.

#### 1-Tosylpiperidin-4-one (**2c**)^35^

The crude product was purified by a direct crystallisation process using* n*-pentane or *n*-hexane as the crystallisation solvent. *R*_*f*_ value: 0.50, Yield: 93% (770 mg); White solid; m.p. 119–121 °C; ^1^H NMR (400 MHz, CDCl_3_) δ 7.70–7.66 (m, 2H), 7.36–7.33 (m, 2H), 3.39 (t, *J* = 6.3 Hz, 4H), 2.53 (t, *J* = 6.3 Hz, 4H),  2.44 (s, 3H); ^13^C NMR (100 MHz, CDCl_3_) δ 205.64, 144.16, 133.38, 129.95, 127.57, 45.89, 40.66, 21.55.

#### 1-((4-(Trifluoromethoxy)phenyl)sulfonyl)piperidin-4-one (**2d**)^50^

The crude product was purified by a direct crystallisation process using* n*-pentane or *n*-hexane as the crystallisation solvent. *R*_*f*_ value: 0.50, Yield: 90% (946 mg); White solid; m.p. 93–95 °C; ^1^H NMR (400 MHz, CDCl_3_) δ 7.90–7.86 (m, 2H), 7.42–7.38 (m, 2H), 3.45 (t, *J* = 6.2 Hz, 4H), 2.59 (t, *J* = 6.2 Hz, 4H); ^13^C NMR (100 MHz, CDCl_3_) δ 205.09, 152.57 (m), 134.94, 129.67 (m), 121.13, 120.21 (q, *J*_*C-F*_ = 259.7 Hz), 45.82, 40.68; ^19^F NMR (377 MHz, CDCl_3_) δ -58.21.

#### 1-((4-(Trifluoromethyl)phenyl)sulfonyl)piperidin-4-one (**2e**)^36^

The crude product was purified by a direct crystallisation process using* n*-pentane or *n*-hexane as the crystallisation solvent. *R*_*f*_ value: 0.50, Yield: 90% (905 mg); White solid; m.p. 134–136 °C; ^1^H NMR (400 MHz, CDCl_3_) δ 7.97–7.92 (m, 2H), 7.86–7.81 (m, 2H), 3.45 (t, *J* = 6.2 Hz, 4H), 2.57 (t, *J* = 6.2 Hz, 4H); ^13^C NMR (100 MHz, CDCl_3_) δ 204.88, 140.34 (m), 134.94 (q, *J*_*C-F*_ = 33.0 Hz), 127.98 (m), 126.53 (m), 123.11 (q, *J*_*C-F*_ = 272.9 Hz), 45.79, 40.69; ^19^F NMR (377 MHz, CDCl_3_) δ -63.70.

#### 1-((3-(Trifluoromethyl)phenyl)sulfonyl)piperidin-4-one (**2f**)^51^

The crude product was purified by a direct crystallisation process using* n*-pentane or *n*-hexane as the crystallisation solvent. *R*_*f*_ value: 0.50, Yield: 92% (920 mg); White solid; m.p. 107–109 °C; ^1^H NMR (400 MHz, CDCl_3_) δ 8.06 (s, 1H), 8.01 (d, *J* = 7.8 Hz, 1H), 7.90 (d, *J* = 7.8 Hz, 1H), 7.73 (t, *J* = 7.8 Hz, 1H), 3.45 (t, *J* = 6.2 Hz, 4H), 2.58 (t, *J* = 6.2 Hz, 4H); ^13^C NMR (100 MHz, CDCl_3_) δ 204.91, 138.09, 132.17 (q, *J*_*C-F*_ = 33.5 Hz), 130.65, 130.24, 129.88 (q, *J*_*C-F*_ = 3.6 Hz), 124.44 (q, *J*_*C-F*_ = 3.9 Hz), 123.09 (q, *J*_*C-F*_ = 273.2 Hz), 45.80, 40.68; ^19^F NMR (377 MHz, CDCl_3_) δ -63.36.

*General procedure for the preparation of tri-OMe-aryl substituted N-sulfonylpiperidine-spiro-1,2,4,5-tetraoxanes (5a–l)* : Preparation of 9-((4-(Trifluoromethoxy)phenyl)sulfonyl)-3-(3,4,5-trimethoxyphenyl)-1,2,4,5-tetraoxa-9-azaspiro[5.5]undecane (**5h**) as a representative:

*Step 1*: 1-((4-(Trifluoromethoxy)phenyl)sulfonyl)piperidin-4-one (**2d,** 0.3 mmol) and H_2_O_2_ (35% solun; 1.5 mmol) were dissolved in two portions of acetonitrile (ACN:10 + 10 mL), subjected to azeotropic distillation (20 + 20 min) on a rotavapour and then left at 25 °C for 18 h. Once the formation of *gem*-dihydroperoxide (DHP; **3d**) was confirmed, the reaction was quenched, and the aqueous phase was extracted with dichloromethane (3 × 10 mL), washed with brine solution (2 × 10 mL) and dried over anhydrous Na_2_SO_4_. Subsequently, the combined organic phase was dried under vacuum.

*Step 2*: **3d** (purified crude mass), **4d** (0.45 mmol), HBF_4_.OEt_2_ (50–55% solun; 25 mol%; 0.075 mmol) and TFE (4.0 mL) were allowed to react at 25 °C for 1 h in a closed reaction vessel. Once the condensation reaction was complete, NaHCO_3_ (10 mL) solution was added dropwise to the reaction mixture as a quenching agent. Then, the aqueous layer was extracted with dichloromethane (3 × 10 mL) and dried over Na_2_SO_4_. Subsequently, the excess solvent from the combined organic phase was removed under vacuum. The resulting crude mass purification was done utilizing column chromatography over silica gel (silica gel: 230–400 mesh; eluent: 10% ethyl acetate: *n*-hexane), and finally, (**5h**) was isolated as a white solid in 73% overall yield.

*General procedure for the one-pot synthesis of C-4 substituted di-/tri-OMe-aryl-spiro-1,2,4,5-tetraoxanes (6a-k)*: Preparation of 9-Methyl-3-(3,4,5-trimethoxyphenyl)-1,2,4,5-tetraoxaspiro[5.5]undecane (**6h**) as a representative:

*Step 1*: A mixture of highly reactive cyclic ketone, i.e. 4-Methylcyclohexan-1-one (**2a**_**2**_, 0.3 mmol) and H_2_O_2_ (1.5 mmol) was dissolved in two portions of acetonitrile (ACN:10 + 10 mL), subjected to azeotropic distillation (20 + 20 min) on a rotavapour and then left at 25 °C for 2 h. **Step 2**: Once the formation of *gem*-dihydroperoxide (DHP; **3a**_**2**_) was confirmed, 3,4,5-*tri*-OMe-aryl-aldehyde (**4d**, 0.45 mmol) and HBF_4_.OEt_2_ (50–55% solun; 25 mol%; 0.075 mmol) and TFE (4.0 mL) were *in-situ* added, and the resulting reaction mixture was further reacted for an additional 1 h at rt. Following reaction completion, quenching of the reaction mixture was done with NaHCO_3_ (10 mL). The aqueous phase was extracted using dichloromethane (3 × 10 mL), washed with brine solution (2 × 10 mL), and dried over anhydrous Na_2_SO_4_. Afterwards, the reaction mixture was subjected to vacuum distillation to remove excess solvent. Finally, the chromatographic purification of the crude mass was done over silica gel (silica gel: 230–400 mesh; eluent: 10% ethyl acetate: *n*-hexane), which afforded 9-Methyl-3-(3,4,5-trimethoxyphenyl)-1,2,4,5-tetraoxaspiro[5.5]undecane (**6h**) as a white solid in 78% overall yield.

*Note*: Both C-4-phenyl substituted 9-Phenyl-3-(2,3,4-trimethoxyphenyl)-1,2,4,5-tetraoxaspiro[5.5]undecane (**6i**) and 9-Phenyl-3-(3,4,5-trimethoxyphenyl)-1,2,4,5-tetraoxaspiro[5.5]undecane (**6j**) analogues were obtained in a chemically acceptable yield (53–67%) via a two-step protocol as described in Scheme 1.

#### 9-(Propylsulfonyl)-3-(2,3,4-trimethoxyphenyl)-1,2,4,5-tetraoxa-9-azaspiro[5.5]undecane (**5a**)

The column chromatography was achieved over silica gel (230–400 mesh) using a 30% ethyl acetate:*n*-hexane mixture as the eluent. *R*_*f*_ value: 0.55, Yield: 24% (50 mg); White solid; m.p. 79–81 °C; ^1^H NMR (400 MHz, CDCl_3_) δ 7.08 (d, *J* = 8.7 Hz, 1H), 6.94 (s, 1H), 6.70 (d, *J* = 8.8 Hz, 1H), 3.97 (s, 3H), 3.89 (s, 3H), 3.88 (s, 3H), 3.52–3.48 (m, 2H), 3.42–3.39 (m, 2H), 2.96–2.90 (m, 2H), 2.71–2.65 (m, 2H), 1.94–1.89 (m, 2H), 1.89–1.84 (m, 2H), 1.08 (t, *J* = 7.4 Hz, 3H); ^13^C NMR (100 MHz, CDCl_3_) δ 156.42, 152.57, 142.14, 123.13, 117.39, 107.34, 105.94, 104.10, 62.02, 60.93, 56.09, 51.74, 42.77, 41.73, 31.73, 30.45, 16.91, 13.14; HRMS (ESI/QTOF) m/z: [M + NH_4_]^+^ Calcd for [C_18_H_27_NO_9_S + NH_4_]^+^; 451.1745; Found 451.1760.

#### 9-(Propylsulfonyl)-3-(3,4,5-trimethoxyphenyl)-1,2,4,5-tetraoxa-9-azaspiro[5.5]undecane (**5b**)

The column chromatography was achieved over silica gel (230–400 mesh) using a 30% ethyl acetate:*n*-hexane mixture as the eluent. *R*_*f*_ value: 0.50, Yield: 26% (55 mg); White solid; m.p. 100–102 °C; ^1^H NMR (400 MHz, CDCl_3_) δ 6.65 (s, 3H), 3.89 (s, 6H), 3.87 (s, 3H), 3.54–3.48 (m, 2H), 3.44–3.39 (m, 2H), 2.97–2.91 (m, 2H), 2.69–2.63 (m, 2H), 1.94–1.90 (m, 2H), 1.90–1.83 (m, 2H), 1.08 (t, *J* = 7.4 Hz, 3H); ^13^C NMR (100 MHz, CDCl_3_) δ 153.59, 140.42, 131.74, 126.14, 108.13, 106.72, 106.33, 104.50, 60.89, 56.26, 51.87, 42.75, 41.68, 31.76, 30.46, 16.92, 13.14; HRMS (ESI/QTOF) m/z: [M + H]^+^ Calcd for [C_18_H_28_NO_9_S]^+^; 434.1480; Found 434.1495; [M + NH_4_]^+^ Calcd for [C_18_H_27_NO_9_S + NH_4_]^+^; 451.1745; Found 451.1760.

#### 9-((4-Methoxyphenyl)sulfonyl)-3-(2,3,4-trimethoxyphenyl)-1,2,4,5-tetraoxa-9-azaspiro[5.5]undecane (**5c**)

The column chromatography was achieved over silica gel (230–400 mesh) using a 35% ethyl acetate:*n*-hexane mixture as the eluent. *R*_*f*_ value: 0.55, Yield: 53% (97 mg); White solid; m.p. 119–121 °C; ^1^H NMR (400 MHz, CDCl_3_) δ 7.74–7.70 (m, 2H), 7.04 (d, *J* = 8.7 Hz, 1H),7.03–6.99 (m, 2H), 6.87 (s, 1H), 6.67 (d, *J* = 8.7 Hz, 1H),, 3.94 (s, 3H), 3.89 (s, 3H), 3.88 (s, 3H), 3.86 (s, 3H), 3.25–3.22 (m, 2H), 3.15–3.12 (m, 2H), 2.68–2.65 (m, 2H), 1.91–1.88 (m, 2H); ^13^C NMR (100 MHz, CDCl_3_) δ 163.19, 156.39, 152.54, 142.11, 129.75, 127.53, 123.06, 117.33, 114.39, 107.29, 105.70, 104.02, 61.99, 60.92, 56.08, 55.64, 43.20, 42.14, 31.10, 29.79; HRMS (ESI/QTOF) m/z: [M + H]^+^ Calcd for [C_22_H_28_NO_10_S]^+^; 498.1429; Found 498.1451.

#### 9-((4-Methoxyphenyl)sulfonyl)-3-(3,4,5-trimethoxyphenyl)-1,2,4,5-tetraoxa-9-azaspiro[5.5]undecane (**5d**)

The column chromatography was achieved over silica gel (230–400 mesh) using a 35% ethyl acetate:*n*-hexane mixture as the eluent. *R*_*f*_ value: 0.50, Yield: 56% (104 mg); White solid; m.p. 149–152 °C; ^1^H NMR (400 MHz, CDCl_3_) δ 7.74–7.71 (m, 2H), 7.04–7.01 (m, 2H), 6.60 (s, 2H), 6.58 (s, 1H), 3.90 (s, 3H), 3.87 (s, 6H), 3.86 (s, 3H), 3.26–3.23 (m, 2H), 3.16–3.13 (m, 2H), 2.66–2.64 (m, 2H), 1.91–1.88 (m, 2H); ^13^C NMR (100 MHz, CDCl_3_) δ 163.23, 153.57, 140.45, 129.74, 127.64, 126.09, 114.41, 108.03, 106.10, 104.49, 60.87, 56.26, 55.65, 43.14, 42.07, 31.16, 29.81; HRMS (ESI/QTOF) m/z: [M + H]^+^ Calcd for [C_22_H_28_NO_10_S]^+^; 498.1429; Found 498.1448.

#### 9-Tosyl-3-(2,3,4-trimethoxyphenyl)-1,2,4,5-tetraoxa-9-azaspiro[5.5]undecane (**5e**)

The column chromatography was achieved over silica gel (230–400 mesh) using a 35% ethyl acetate:*n*-hexane mixture as the eluent. *R*_*f*_ value: 0.50, Yield: 47% (45 mg); White solid; m.p. 138–140 °C; ^1^H NMR (400 MHz, CDCl_3_) δ 7.66–7.63 (m, 2H), 7.34–7.31 (m, 2H), 7.02 (d, *J* = 8.7 Hz, 1H),, 6.85 (s, 1H), 6.65 (d,  *J* = 8.8 Hz, 1H), 3.92 (s, 3H), 3.86 (s, 3H), 3.84 (s, 3H), 3.24–3.21 (m, 2H), 3.14–3.11 (m, 2H), 2.66–2.63 (m, 2H), 2.43 (s, 3H), 1.89–1.86 (m, 2H); ^13^C NMR (100 MHz, CDCl_3_) δ 156.30, 152.45, 143.81, 142.03, 132.94, 129.69, 127.56, 122.97, 117.24, 107.17, 105.61, 103.93, 61.77, 60.72, 56.08, 43.09, 42.03, 31.03, 29.71, 21.45; HRMS (ESI/QTOF) m/z: [M + H]^+^ Calcd for [C_22_H_28_NO_9_S]^+^; 482.1480; Found 482.1517; [M + NH_4_]^+^ Calcd for [C_22_H_27_NO_9_S + NH_4_]^+^; 499.1745; Found 499.1783.

#### 9-Tosyl-3-(3,4,5-trimethoxyphenyl)-1,2,4,5-tetraoxa-9-azaspiro[5.5]undecane (5f)

The column chromatography was achieved over silica gel (230–400 mesh) using a 35% ethyl acetate:*n*-hexane mixture as the eluent. *R*_*f*_ value: 0.55, Yield: 58% (89 mg); White solid; m.p. 147–149 °C; ^1^H NMR (400 MHz, CDCl_3_) δ 7.67–7.64 (m, 2H), 7.34–7.32 (m, 2H), 6.58 (s, 2H), 6.56 (s, 1H), 3.85 (s, 6H), 3.84 (s, 3H), 3.25–3.22 (m, 2H), 3.15–3.12 (m, 2H), 2.65–2.62 (m, 2H), 2.44 (s, 3H), 1.89–1.86 (m, 2H); ^13^C NMR (100 MHz, CDCl_3_) δ 153.56, 143.95, 140.43, 133.07, 129.86, 127.65, 126.08, 108.04, 106.08, 104.46, 60.87, 56.25, 43.14, 42.06, 31.16, 29.82, 21.55; HRMS (ESI/QTOF) m/z: [M + H]^+^ Calcd for [C_22_H_28_NO_9_S]^+^; 482.1480; Found 482.1529; [M + NH_4_]^+^ Calcd for [C_22_H_27_NO_9_S + NH_4_]^+^; 499.1745; Found 499.1795.

#### 9-((4-(Trifluoromethoxy)phenyl)sulfonyl)-3-(2,3,4-trimethoxyphenyl)-1,2,4,5-tetraoxa-9-azaspiro[5.5]undecane (**5g**)

The column chromatography was achieved over silica gel (230–400 mesh) using a 35% ethyl acetate:*n*-hexane mixture as the eluent. *R*_*f*_ value: 0.55, Yield: 61% (125 mg); White solid; m.p. 98–101 °C; ^1^H NMR (400 MHz, CDCl_3_) δ 7.84–7.81 (m, 2H), 7.38–7.35 (m, 2H), 7.02 (d, *J* = 8.7 Hz, 1H), 6.86 (s, 1H), 6.66 (d, *J* = 8.7 Hz, 1H), 3.93 (s, 3H), 3.87 (s, 3H), 3.84 (s, 3H), 3.28–3.25 (m, 2H), 3.18–3.15 (m, 2H), 2.69–2.66 (m, 2H), 1.92–1.89 (m, 2H); ^13^C NMR (100 MHz, CDCl_3_) δ 156.48, 152.61, 152.48 (m), 142.22, 134.79, 129.73, 123.04, 121.03, 120.26 (q, *J*_*C-F*_ = 260.9 Hz), 117.32, 107.37, 105.55, 104.16, 61.99, 60.91, 56.10, 43.18, 42.10, 31.19, 29.89; ^19^F NMR (377 MHz, CDCl_3_) δ -58.18; HRMS (ESI/QTOF) m/z: [M + H]^+^ Calcd for [C_22_H_25_F_3_NO_10_S]^+^; 552.1146; Found 552.1185; [M + NH_4_]^+^ Calcd for [C_22_H_24_F_3_NO_10_S + NH_4_]^+^; 569.1411; Found 569.1446.

#### 9-((4-(Trifluoromethoxy)phenyl)sulfonyl)-3-(3,4,5-trimethoxyphenyl)-1,2,4,5-tetraoxa-9-azaspiro[5.5]undecane (**5h**)

The column chromatography was achieved over silica gel (230–400 mesh) using a 30% ethyl acetate:*n*-hexane mixture as the eluent. *R*_*f*_ value: 0.50, Yield: 73% (150 mg); White solid; m.p. 124–126 °C; ^1^H NMR (400 MHz, CDCl_3_) δ 7.85–7.81 (m, 2H), 7.39–7.36 (m, 2H), 6.59 (s, 2H), 6.58 (s, 1H), 3.86 (s, 6H), 3.84 (s, 3H), 3.29–3.27 (m, 2H), 3.19–3.16 (m, 2H), 2.68–2.65 (m, 2H), 1.93–1.90 (m, 2H); ^13^C NMR (100 MHz, CDCl_3_) δ 153.63, 152.51 (m), 140.62, 134.81, 129.73, 126.01, 121.05, 120.26 (q, *J*_C-F_ = 259.9 Hz),118.97, 108.11, 105.94, 104.58, 60.88, 56.29, 43.13, 42.03, 31.23, 29.90; ^19^F NMR (377 MHz, CDCl_3_) δ -58.18; HRMS (ESI/QTOF) m/z: [M + H]^+^ Calcd for [C_22_H_25_F_3_NO_10_S]^+^; 552.1146; Found 552.1174; [M + NH_4_]^+^ Calcd for [C_22_H_24_F_3_NO_10_S + NH_4_]^+^; 569.1411; Found 569.1440.

#### 9-((4-(Trifluoromethyl)phenyl)sulfonyl)-3-(2,3,4-trimethoxyphenyl)-1,2,4,5-tetraoxa-9-azaspiro[5.5]undecane (**5i**)

The column chromatography was achieved over silica gel (230–400 mesh) using a 25% ethyl acetate:*n*-hexane mixture as the eluent. *R*_*f*_ value: 0.65, Yield: 51% (88 mg); White solid; m.p. 98–100 °C; ^1^H NMR (400 MHz, CDCl_3_) δ 7.94–7.91 (m, 2H), 7.85–7.82 (m, 2H), 7.03 (d, *J* = 8.7 Hz, 1H), 6.88 (s, 1H), 6.68 (d, *J* = 8.7 Hz, 1H), 3.94 (s, 3H), 3.88 (s, 3H), 3.86 (s, 3H), 3.31–3.28 (m, 2H), 3.22–3.18 (m, 2H), 2.72–2.67 (m, 2H), 1.94–1.91 (m, 2H); ^13^C NMR (100 MHz, CDCl_3_) δ 156.45, 152.56, 142.13, 139.91, 134.75 (q, *J*_*C-F*_ = 33.0 Hz), 128.07, 126.44 (q, *J*_*C-F*_ = 3.7 Hz), 123.17 (q, *J*_*C-F*_ = 273.2 Hz), 123.17, 117.21, 107.29, 105.45, 104.13, 61.99, 60.92, 56.08, 43.19, 42.09, 31.14, 29.84; ^19^F NMR (377 MHz, CDCl_3_) δ -63.67; HRMS (ESI/QTOF) m/z: [M + NH_4_]^+^ Calcd for [C_22_H_24_F_3_NO_9_S + NH_4_]^+^; 553.1462; Found 553.1488.

#### 9-((4-(Trifluoromethyl)phenyl)sulfonyl)-3-(3,4,5-trimethoxyphenyl)-1,2,4,5-tetraoxa-9-azaspiro[5.5]undecane (**5j**)

The column chromatography was achieved over silica gel (230–400 mesh) using a 25% ethyl acetate:*n*-hexane mixture as the eluent. *R*_*f*_ value: 0.60, Yield: 72% (125 mg); White solid; m.p. 155–157 °C; ^1^H NMR (400 MHz, CDCl_3_) δ 7.94–7.91 (m, 2H), 7.85–7.82 (m, 2H), 6.60 (s, 2H),6.59 (s, 1H), 3.87 (s, 6H), 3.86 (s, 3H), 3.34–3.28 (m, 2H), 3.24–3.18 (m, 2H), 2.71–2.64 (m, 2H), 1.96–1.90 (m, 2H); ^13^C NMR (100 MHz, CDCl_3_) δ 153.58, 140.44, 139.93 (m), 134.78 (q, *J*_*C-F*_ = 32.9 Hz), 128.07, 126.45 (q, *J*_*C-F*_ = 3.7 Hz), 125.97, 123.16 (q, *J*_*C-F*_ = 273.2 Hz), 108.10, 105.84, 104.42, 60.88, 56.24, 43.14, 42.03, 31.17, 29.85; ^19^F NMR (377 MHz, CDCl_3_) δ -63.67; HRMS (ESI/QTOF) m/z: [M + H]^+^ Calcd for [C_22_H_25_F_3_NO_9_S]^+^; 536.1197; Found 536.1219; [M + NH_4_]^+^ Calcd for [C_22_H_24_F_3_NO_9_S + NH_4_]^+^; 553.1462; Found 553.1488.

#### 9-((3-(Trifluoromethyl)phenyl)sulfonyl)-3-(2,3,4-trimethoxyphenyl)-1,2,4,5-tetraoxa-9-azaspiro[5.5]undecane (**5k**)

The column chromatography was achieved over silica gel (230–400 mesh) using a 30% ethyl acetate:*n*-hexane mixture as the eluent. *R*_*f*_ value: 0.65, Yield: 60% (42 mg); White solid; m.p. 110–112 °C; ^1^H NMR (400 MHz, CDCl_3_) δ 8.06 (s, 1H), 7.98 (d, *J* = 7.9 Hz, 1H), 7.90 (d, *J* = 7.9 Hz, 1H), 7.73 (t, *J* = 7.8 Hz, 1H), 7.04 (d, *J *= 8.7 Hz, 1H),6.88 (s, 1H), 6.68 (d, *J* = 8.8 Hz,  1H), 3.94 (s, 3H), 3.89 (s, 3H), 3.86 (s, 3H), 3.32–3.29 (m, 2H), 3.23–3.19 (m, 2H), 2.73–2.69 (m, 2H), 1.96–1.92 (m, 2H); ^13^C NMR (100 MHz, CDCl_3_) δ 156.46, 152.56, 142.14, 137.67, 132.05 (q, *J*_*C-F*_ = 33.5 Hz), 130.69, 130.10, 129.72 (q, *J*_*C-F*_ = 3.5 Hz), 124.54 (q, *J*_C-F_ = 3.8 Hz), 123.14 (q), 123.04, 117.22, 107.32, 105.47, 104.12, 61.99, 60.92, 56.09, 43.16, 42.09, 31.13, 29.84; ^19^F NMR (377 MHz, CDCl_3_) δ -63.33; HRMS (ESI/QTOF) m/z: [M + H]^+^ Calcd for [C_22_H_25_F_3_NO_9_S]^+^; 536.1197; Found 536.1172; [M + NH_4_]^+^ Calcd for [C_22_H_24_F_3_NO_9_S + NH_4_]^+^; 553.1462; Found 553.1439.

#### 9-((3-(Trifluoromethyl)phenyl)sulfonyl)-3-(3,4,5-trimethoxyphenyl)-1,2,4,5-tetraoxa-9-azaspiro[5.5]undecane (**5l**)

The column chromatography was achieved over silica gel (230–400 mesh) using a 30% ethyl acetate:*n*-hexane mixture as the eluent. *R*_*f*_ value: 0.65, Yield: 78% (205 mg); White solid; m.p. 123–125 °C; ^1^H NMR (400 MHz, CDCl_3_) δ 8.04 (s, 1H), 7.97 (d, *J* = 7.9 Hz, 1H), 7.89 (d, *J *= 7.8 Hz, 1H), 7.71 (t, *J* = 7.9 Hz, 1H), 6.59 (s, 2H)6.57 (s, 1H), 3.86 (s, 6H), 3.84 (s, 3H), 3.32–3.28 (m, 2H), 3.22–3.18 (m, 2H), 2.69–2.65 (m, 2H), 1.94–1.90 (m, 2H); ^13^C NMR (100 MHz, CDCl_3_) δ 153.59, 140.49, 137.72, 132.06 (q, *J*_*C-F*_ = 33.5 Hz), 130.69, 130.13, 129.76 (q, *J*_*C-F*_ = 3.6 Hz), 125.97, 124.54 (q, *J*_*C-F*_ = 3.2 Hz), 123.14 (q, *J*_*C-F*_ = 273.1 Hz), 108.12, 105.85, 104.47, 60.89, 56.26, 43.11, 42.02, 31.17, 29.86; ^19^F NMR (377 MHz, CDCl_3_) δ -63.32; HRMS (ESI/QTOF) m/z [M + H]^+^ Calcd for [C_22_H_25_F_3_NO_9_S]^+^; 536.1197; Found 536.1175; [M + NH_4_]^+^ Calcd for [C_22_H_24_F_3_NO_9_S + NH_4_]^+^; 553.1462; Found 553.1441.

#### 3,3-dimethyl-6-(3,4,5-trimethoxyphenyl)-1,2,4,5-tetraoxane (**6k**)

The column chromatography was achieved over silica gel (230–400 mesh) using a 10% ethyl acetate:*n*-hexane mixture as the eluent. *R*_*f*_ value: 0.50, Yield: 19% (95 mg); White solid; m.p. 84–86 °C; ^1^H NMR (400 MHz, CDCl_3_) δ 6.69 (s, 2H), 6.60 (s, 1H), 3.90 (s, 6H), 3.87 (s, 3H), 1.98 (s, 3H), 1.45 (s, 3H); ^13^C NMR (100 MHz, CDCl_3_) δ 153.50, 140.19, 126.65, 108.35, 107.58, 104.56, 60.87, 56.21, 22.45, 21.35; HRMS (ESI/QTOF) m/z [M + Na]^+^ Calcd for [C_13_H_18_O_7_ + Na]^+^; 309.0945; Found 309.0943.

*Note*: ^1^H, ^13^C{^1^H} NMR characterization data for compounds (**6a–j**) were compared and are available in our recently published article^38^.

#### In vitro antiplasmodial assay on *Plasmodium falciparum*^38^

The chloroquine-resistant strain FcB-1 of *P. falciparum* from Colombia was continuously maintained in vitro on O^+^ human erythrocytes provided by the Etablissement Français du Sang in RPMI-1640 medium supplemented with 0.5% Albumax-II, at 37 °C, under 5% CO_2_ as described^52^. Parasites were routinely synchronized using 5% *D*-sorbitol treatment^53^. Antiplasmodial assays were performed in 96-well plates as previously described^54^. Briefly, stock solutions of compounds were prepared in pure DMSO. They were serially diluted two-fold in the plate with 100 µL of culture medium per well. Then, 100 µL of ring-stage synchronized parasite culture at 1% parasitemia and 2% hematocrit were added per well. Artesunate was used as a positive control. DMSO was used as a negative control, and no effect on parasite growth was measured up to 0.5%. After 48 h of incubation at 37 °C, under 5% CO_2_, plates were frozen at − 20 °C. After thawing, well contents were mixed and 100 µL transferred to a new plate and 80 μL of lysis buffer (20 mM Tris at pH 7.4, 5 mM EDTA, 0.008% (wt/vol) saponin, and (0.08% (vol/vol) Triton X-100) were added to each well. Plates were incubated for 20 min, and autofluorescence values were measured in a BioTek Cytation 1 cell imaging multimode reader (Agilent™), with excitation and emission wavelength bands at 485/20 and 528/20 nm (510 nm cut-off), respectively. Then, 20 μL of lysis buffer containing Thiazole Green (SYBR® Green I, Biotium) was added to each well, and plates were incubated for an additional 40 min at 37 °C before fluorescence recording. Autofluorescence values were subtracted from the Thiazole Green fluorescence values. 100% growth inhibition was obtained from the artesunate treatment. The drug concentration that causes 50% inhibition of parasite growth (IC_50_) was determined using the IC50 calculator software from AAT Bioquest (https://www.aatbio.com/tools/ic50-calculator). The index of selectivity was defined as the ratio of the IC_50_ value on “normal/human cancer” cells to that of *P*. *falciparum*^55^.

#### Cytotoxicity study^38^

In vitro cultivation and maintenance of culture^38^:

Assays on the cervical carcinoma cell line HeLa (ATCC) were performed in 96-well plates using the resazurin assay as described^52^. Briefly, cells were seeded at 2000 cells per well in 100 µL RPMI 1640 medium containing 10% fetal calf serum. After 24 h at 37 °C and under 5% CO_2_, cells were washed, and twofold dilutions of the drug in culture medium were added (200 µL per well). The final DMSO concentration in the culture remained below 0.5%. The cultures were incubated at 37 °C under 5% CO_2_ for 3 days. Untreated cultures were included as controls. The cytotoxicity was determined by adding resazurin (45 µM final solution) and measuring fluorescence after 4 h of incubation, at λ excitation 528 nm and λ emission 590 nm. The percentage of inhibition of cell growth was calculated by comparing the fluorescence of cells maintained in the presence of the drug to that in the absence of the drug. The concentrations causing 50% (LD_50_) of cell growth inhibition were obtained from the drug concentration–response curves using the IC_50_ calculator software from AAT Bioquest (https://www.aatbio.com/tools/ic50-calculator).

## Cytotoxicity studies^38^

### Cell culture and evaluation of cytotoxic effect^38^

#### Reagents

The following reagents, fetal bovine serum (FBS), penicillin–streptomycin-L-glutamine solution, Dulbecco’s phosphate-buffered saline (DPBS), trypsin–EDTA, dimethyl sulfoxide (DMSO), 3-(4, 5-dimethylthiazol-2-yl)-2,5-diphenyltetrazolium bromide (MTT) were purchased from Sigma Aldrich (St. Louis, MO, USA, USA). The Roswell Park Memorial Institute (RPMI) 1640 Medium was purchased from Gibco (Thermo Fisher Scientific, Waltham, MA, USA). The DMSO used for the MTT assay was obtained from Avantor Performance Materials (Gliwice, Poland).

#### Cell culture

The human ovarian carcinoma A2780 cells (AddexBio Technologies, San Diego, CA, USA) were maintained in RPMI medium supplemented with 10% (*v*/*v*) fetal bovine serum (FBS) and 1% (*v*/*v*) of a solution containing *L*-glutamine (200 mM), penicillin (10,000 units), and streptomycin (10 mg/mL). Cells were cultured under standard conditions (37 °C, 5% CO_2_).

#### Tested compounds

Novel sulonylopiperidone peroxides were dissolved in DMSO at a concentration of 10 mM. The stock solutions were stored at − 20 °C.

#### MTT assay

For cytotoxicity assessment, A2780 cells were seeded at a density of 10,000 cells per well in 96-well plates and allowed to adhere for 24 h. Cells were then treated with the tested compounds at concentrations of 10 µM and 1 µM for 72 h. DMSO (0.1% *v*/*v* in culture medium) served as the vehicle control. Cell viability was determined using the MTT assay. After incubation with the tested compound, the A2780 cells were washed with DPBS. Then, MTT solution (5 mg/mL in DPBS) was added to the culture medium (20 µL to 150 µL) to achieve a final volume of 170 µL per well. After incubation for 1.5 h under standard culture conditions, formazan crystals were dissolved by adding 200 µL of DMSO. Absorbance was measured at 570 nm using a microplate reader (Biotek Instruments, Elx-800, Winooski, VT, USA).

## Supplementary Information

Supplementary Information.

## Data Availability

The data underlying this study are available in the published article and its Supporting Information.
