# Supplementary material for: Efficient and metal catalyst-free approach towards highly-substituted N-sulfonylpiperidine-/mono-spiro-1,2,4,5-tetraoxanes for potential applications in chemotherapy
Source: Sci Rep. 2026 May 25;16:23909. doi: 10.1038/s41598-026-48982-6 (PMC13434043; doi:10.1038/s41598-026-48982-6)
Supplement: Supplementary file 1 [file 41598_2026_48982_MOESM1_ESM.docx]

**Supporting Information
Efficient and Metal Catalyst-free Approach Towards Highly-substituted N-sulfonylpiperidine-/mono-spiro-1,2,4,5-tetraoxanes for Potential Applications in Chemotherapy**

Mohit K. Tiwari,^a,^* Malgorzata Kucinska,^b^ Agnieszka Zgoła-Grześkowiak,^c^ Marek Murias,^b^ Philippe Grellier,^d^ Łukasz Marczak,^e^ Lukasz Popenda ^f^ and Tomasz Goslinski ^a^

^a^Poznan University of Medical Sciences, Chair and Department of Chemical Technology of Drugs, ul. Rokietnicka 3, 60-806, Poznań, Poland.

^b^Poznan University of Medical Sciences, Chair and Department of Toxicology, ul. Rokietnicka 3, 60-806, Poznań, Poland

^c^Institute of Chemistry and Technical Electrochemistry, Poznan University of Technology, Berdychowo 4, 60-965, Poznań, Poland

^d^UMR7245 MCAM, CNRS, National Museum of Natural History, CP 52, 57 rue Cuvier, 75005 Paris, France

^e^Institute of Bioorganic Chemistry, Polish Academy of Sciences, Noskowskiego 12/14, 61-704 Poznań, Poland.

^f^NanoBioMedical Centre, Adam Mickiewicz University, Wszechnicy Piastowskiej 3, 61-614 Poznań, Poland.

| **Contents** | **Page No.** |
| --- | --- |
| 1. MS and UV purity analysis of compounds (Scheme **5a-l** and **6k**). | S2-S53 |
| 2. Characterization spectra (^1^H, ^13^C, ^19^F NMR) of **2a**-**f** and **5a-l** and **6k** | S54-S81 |

**E-mail:* [*mtiwari@ump.edu.pl*](mailto:mtiwari@ump.edu.pl)

**To be published on the Web.**

**1. Confirmation of structure and purity by mass spectra and chromatographic analysis:**

The structure and purity of synthesized 1,2,4,5-tetraoxanes (**5a-5l**) were confirmed. The HPLC-UV chromatograms of synthesized compounds showed only one peak in the chromatograms recorded at 254 nm and two absorption wavelength maxima. Peak purity was also confirmed (Scheme **5a-5l**).

In the MS analyses, not only protonated molecules [M+H]^+^ were observed, but also adducts with sodium ions [M+Na]^+^, potassium ions [M+K]^+^ or ammonium ions [M+NH_4_]^+^ were found as well as dimers with the same ions. Strong fragmentation was found in the electrospray source for all of these compounds. Collision-induced fragmentation was obtained in the spectrometer, and ions arose mainly from the cleavage of the tetraoxane ring (Scheme **5a-5l**).

**Scheme 5a: Peak purity by UV and mass spectra of compound 5a.**

**HPLC-UV chromatograms:**

The peak purity and chromatograms at two absorption maxima and at λ = 254 nm, retention time = 4.24 min

**Scheme 5a (continued):**

**Low-resolution mass spectrum:**

Important ions were detected at m/z 434 [M+H]^+^, 451 [M+NH_4_]^+^, 456 [M+Na]^+^, 472 [M+K]^+^, 889 [2M+Na]^+^and from the in-source fragmentation at m/z 238, 180, 153. Collision-induced fragmentation of the ion at m/z 451 forms ions at m/z 434, 238, 185, 184, 180, 153.

**Scheme 5a (continued):**

**Fragmentation scheme:**

**Scheme 5a (continued):**

**High-resolution mass spectrum:**

The ions were detected at m/z 451.1760 [M+NH_4_]^+^ and 889.2734 [2M+Na]^+^. Characteristic fragments were found at m/z 238.0747, 180.0685, 153.0540, confirming low-resolution MS results.

**Scheme 5b: Peak purity by UV and mass spectra of compound 5b.**

**HPLC-UV chromatograms:**

The peak purity and chromatograms at two absorption maxima and at λ = 254 nm, retention time = 3.79 min; an impurity peak was noted at retention time = 3.19 min (UV spectrum included).

**Scheme 5b (continued):**

**Low-resolution mass spectrum:**

Important ions were detected at m/z 434 [M+H]^+^, 451 [M+NH_4_]^+^, 456 [M+Na]^+^, 472 [M+K]^+^, 889 [2M+Na]^+^and from the in-source fragmentation at m/z 185, 180, 153. Collision-induced fragmentation of the ion at m/z 434 forms ions at m/z 238, 212, 185, 184, 180, 153.

**Scheme 5b (continued):**

**Fragmentation scheme:**

**Scheme 5b (continued):**

**High-resolution mass spectrum:**

The ions were detected at m/z 434.1495 [M+H]^+^, 451.1760 [M+NH_4_]^+^, and 889.2734 [2M+Na]^+^. Characteristic fragments were found at m/z 238.0746, 212.0677, 180.0684, 153.0538, confirming low-resolution MS results.

**Scheme 5c: Peak purity by UV and mass spectra of compound 5c.**

**HPLC-UV chromatograms:**

The peak purity and chromatograms at two absorption maxima and at λ = 254 nm, retention time = 4.86 min; an impurity peak was noted at retention time = 4.23 min (UV spectrum included).

**Scheme 5c (continued):**

**Low-resolution mass spectrum:**

Important ions were detected at m/z 498 [M+H]^+^, 515 [M+NH_4_]^+^, 520 [M+Na]^+^, and from the in-source fragmentation at m/z 244, 185, 184, 153. Collision-induced fragmentation of the ion at m/z 498 forms ions at m/z 244, 185, 184, 153.

**Scheme 5c (continued):**

**Fragmentation scheme:**

**Scheme 5c (continued):**

**High-resolution mass spectra:**

The ions were detected at m/z 498.1451 [M+H]^+^, m/z 995.2822 [2M+H]^+^, 1017.2645 [2M+Na]^+^. Characteristic fragments were found at m/z 244.0643 and 153.0541.

**Scheme 5d: Peak purity by UV and mass spectra of compound 5d.**

**HPLC-UV chromatograms:**

The peak purity and chromatograms at two absorption maxima and at λ = 254 nm, retention time = 4.35 min.

**Scheme 5d (continued):**

**Low-resolution mass spectrum:**

Important ions were detected at m/z 498 [M+H]^+^, 515 [M+NH_4_]^+^, 520 [M+Na]^+^, 337 [M+K]^+^, 995 [2M+H]^+^, 1017 [2M+Na]^+^ and from the in-source fragmentation at m/z 244, 212, 185, 153. Collision-induced fragmentation of the ion at m/z 498 forms ions at m/z 244, 212, 185, 184, 153.

**Scheme 5d (continued):**

**Fragmentation scheme:**

**Scheme 5d (continued):**

**High-resolution mass spectrum:**

The ions were detected at m/z 498.1448 [M+H]^+^, m/z 995.2816 [2M+H]^+^, 1012.3081 [2M+NH_4_]^+^. Characteristic fragments were found at m/z 244.0639, 212.0679, 184.0728, 153.0538 confirming low-resolution MS results.

**Scheme 5e: Peak purity by UV and mass spectra of compound 5e.**

**HPLC-UV chromatograms:**

The peak purity and chromatograms at two absorption maxima and at λ = 254 nm, retention time = 5.19 min.

**Scheme 5e (continued):**

**Low-resolution mass spectrum:**

Important ions were detected at m/z 482 [M+H]^+^, 499 [M+NH_4_]^+^, 504 [M+Na]^+^, 520 [M+K]^+^, 985 [2M+Na]^+^ and from the in-source fragmentation at m/z 286, 228, 184, 153. Collision-induced fragmentation of the ion at m/z 569 forms ions at m/z 286, 228, 185, 184, 153.

**Scheme 5e (continued):**

**Fragmentation scheme:**

**Scheme 5e (continued):**

**High-resolution mass spectra:**

The ions were detected at 482.1517 [M+H]^+^, 499.1783 [M+NH_4_]^+^, 985.2781 [2M+Na]^+^. Characteristic fragments were found at m/z 286.0758, 228.0698, 184.0734, 153.0545 confirming low-resolution MS results.

**Scheme 5f: Peak purity by UV and mass spectra of compound 5f.**

**HPLC-UV chromatograms:**

The peak purity and chromatograms at two absorption maxima and at λ = 254 nm, retention time = 4.65 min.

**Scheme 5f (continued):**

**Low-resolution mass spectra:**

Important ions were detected at m/z 482 [M+H]^+^, 499 [M+NH_4_]^+^, 504 [M+Na]^+^, 520 [M+K]^+^ and from the in-source fragmentation at m/z 228, 185, 184, 153. Collision-induced fragmentation of the ion at m/z 499 forms ions at m/z 228, 185, 184, 153.

**Scheme 5f (continued):**

**Fragmentation scheme:**

**Scheme 5f (continued):**

**High-resolution mass spectra:**

The ions were detected at m/z 482.1529, [M+H]^+^, 499.1795 [M+NH_4_]^+^, 985.2806 [2M+Na]^+^. Characteristic fragments were found at m/z 228.0704, 185.0818, 184.0739 confirming low-resolution MS results.

**Scheme 5g: Peak purity by UV and mass spectra of compound 5g.**

**HPLC-UV chromatograms:**

The peak purity and chromatograms at two absorption maxima and at λ = 254 nm, retention time = 5.46 min

**Scheme 5g (continued):**

**Low-resolution mass spectrum:**

Important ions were detected at m/z 552 [M+H]^+^, 569 [M+Na]^+^, 574 [M+Na]^+^, 1125 [2M+Na]^+^ and from the in-source fragmentation at m/z 356, 298, 185, 184, 153. Collision-induced fragmentation of the ion at m/z 569 forms ions at m/z 356, 298, 185, 184, 153.

**Scheme 5g (continued):**

**Fragmentation scheme:**

**Scheme 5g (continued):**

**High-resolution mass spectrum:**

The ions were detected at m/z 552.1185 [M+H]^+^, 569.1446 [M+NH_4_]^+^, 1125.2104 [2M+Na]^+^. Characteristic fragments were found at m/z 356.0430, 298.0370, 184.0731, 153.0544 confirming low-resolution MS results.

**Scheme 5h: Peak purity by UV and mass spectra of compound 5h.**

**HPLC-UV chromatograms:**

The peak purity and chromatograms at two absorption maxima and at λ = 254 nm, retention time = 4.96 min

**Scheme 5h (continued):**

**Low-resolution mass spectrum:**

Important ions were detected at m/z 552 [M+H]^+^, 569 [M+Na]^+^, 574 [M+Na]^+^, 1125 [2M+Na]^+^ and from the in-source fragmentation at m/z 356, 298, 185, 153. Collision-induced fragmentation of the ion at m/z 552 forms ions at m/z 356, 298, 212, 185, 184, 153.

**Scheme 5h (continued):**

**Fragmentation scheme:**

**Scheme 5h (continued):**

**High-resolution mass spectrum:**

The ions were detected at m/z 552.1174 [M+H]^+^, 569.1440 [M+NH_4_]^+^, 1125.2090 [2M+Na]^+^. Characteristic fragments were found at m/z 356.0426, 298.0365, 212.0679, 185.0805, 153.0539 confirming low-resolution MS results.

**Scheme 5i: Peak purity by UV and mass spectra of compound 5i.**

**HPLC-UV chromatograms:**

The peak purity and chromatograms at two absorption maxima and at λ = 254 nm, retention time = 5.34 min.

**Scheme 5i (continued):**

**Low-resolution mass spectrum:**

Important ions were detected at m/z 536 [M+H]^+^, 553 [M+NH_4_]^+^, 558 [M+Na]^+^ and from the in-source fragmentation at m/z 340, 282, 185, 153. Collision-induced fragmentation of the ion at m/z 536 forms ions at m/z 340, 282, 185, 184, 153.

**Scheme 5i (continued):**

**Fragmentation scheme:**

**Scheme 5i (continued):**

**High-resolution mass spectrum:**

The ions were detected at m/z 553.1488 [M+NH_4_]^+^, 1093.2187 [2M+Na]^+^. Characteristic fragments were found at m/z 340.0472, 282.0412, 185.0805, 153.0539 confirming low-resolution MS results.

**Scheme 5j: Peak purity by UV and mass spectra of compound 5j.**

**HPLC-UV chromatograms:**

The peak purity and chromatograms at two absorption maxima and at λ = 254 nm, retention time = 4.83 min.

**Scheme 5j (continued):**

**Low-resolution mass spectrum:**

Important ions were detected at m/z 536 [M+H]^+^, 553 [M+NH_4_]^+^, 558 [M+Na]^+^ and from the in-source fragmentation at m/z 340, 282, 185, 153. Collision-induced fragmentation of the ion at m/z 536 forms ions at m/z 340, 282, 212, 185, 184, 153.

**Scheme 5j (continued):**

**Fragmentation scheme:**

**Scheme 5j (continued):**

**High-resolution mass spectrum:**

The ions were detected at m/z 536.1219 [M+H]^+^, 553.1488 [M+NH_4_]^+^, 1093.2187 [2M+Na]^+^. Characteristic fragments were found at m/z 340.0475, 282.0412, 212.0681, 185.0807, 153.0539 confirming low-resolution MS results.

**Scheme 5k: Peak purity by UV and mass spectra of compound 5k.**

**HPLC-UV chromatograms:**

The peak purity and chromatograms at two absorption maxima and at λ = 254 nm, retention time = 5.41 min; an impurity peak was noted at retention time = 4.78 min (UV spectrum included).

**Scheme 5k (continued):**

**Low-resolution mass spectrum:**

Important ions were detected at m/z 536 [M+H]^+^, 553 [M+NH_4_]^+^, 558 [M+Na]^+^, 1093 [2M+Na]^+^ and from the in-source fragmentation at m/z 340, 282, 185, 153. Collision-induced fragmentation of the ion at m/z 536 forms ions at m/z 340, 282, 185, 184, 153.

**Scheme 5k (continued):**

**Fragmentation scheme:**

**Scheme 5k (continued):**

**High-resolution mass spectrum:**

The ions were detected at m/z 536.1172 [M+H]^+^, 553.1439 [M+NH_4_]^+^, 1093.2083 [2M+Na]^+^. Characteristic fragments were found at m/z 340.0445 282.0391, 185.0800, 153.0539 confirming low-resolution MS results.

**Scheme 5l: Peak purity by UV and mass spectra of compound 5l.**

**HPLC-UV chromatograms:**

The peak purity and chromatograms at two absorption maxima and at λ = 254 nm, retention time = 4.88 min.

**Scheme 5l (continued):**

**Low-resolution mass spectrum:**

Important ions were detected at m/z 536 [M+H]^+^, 553 [M+NH_4_]^+^, 558 [M+Na]^+^, 574 [M+K]^+^ 1088 [M+NH_4_]^+^, 1093 [M+Na]^+^, 1109 [M+K]^+^ and from the in-source fragmentation at m/z 340, 282, 185, 153. Collision-induced fragmentation of the ion at m/z 536 forms ions at m/z 340, 282, 212, 185, 184, 153.

**Scheme 5l (continued):**

**Fragmentation scheme:**

**Scheme 5l (continued):**

**High-resolution mass spectra:**

The ions were detected at m/z 536.1175 [M+H]^+^, 553.1441 [M+NH_4_]^+^. Characteristic fragments were found at m/z 282.0395, 212.0671, 185.0803, 153.0544 confirming low-resolution MS results.

**Scheme 6k: Peak purity by UV and mass spectra of compound 6k.**

**HPLC-UV chromatograms:**

The peak purity and chromatograms at two absorption maxima and at λ = 254 nm, retention time = 4.95 min

**Scheme 6k (continued):**

**Low-resolution mass spectrum:**

Important ions were detected at m/z 287 [M+H]^+^, 304 [M+NH_4_]^+^, 309 [M+Na]^+^, 325 [M+K]^+^, 590 [2M+NH_4_]^+^, 595 [2M+Na]^+^, 611 [2M+K]^+^. Collision-induced fragmentation of the ion at m/z 287 forms ions at m/z 212, 196, 185, 184, 169, 153.

**Scheme 6k (continued):**

**Fragmentation scheme:**

**Scheme 6k (continued):**

**High-resolution mass spectrum:**

The ions were detected at m/z 309.0943 [M+Na]^+^ and 595.1995 [2M+Na]^+^.

**2. Characterization spectra (^1^H, ^13^C, ^19^F NMR) of 2a**-**f** and **5a-l and 6k.**

^1^H NMR spectrum of compound **2a**

^13^C NMR spectrum of compound **2a**

^1^H NMR spectrum of compound **2b**

^13^C NMR spectrum of compound **2b**

^1^H NMR spectrum of compound **2c**

^13^C NMR spectrum of compound **2c**

^1^H NMR spectrum of compound **2d**

^13^C NMR spectrum of compound **2d**

^19^F NMR spectrum of compound **2d**

^1^H NMR spectrum of compound **2e**

^13^C NMR spectrum of compound **2e**

^19^F NMR spectrum of compound **2e**

^1^H NMR spectrum of compound **2f**

^13^C NMR spectrum of compound **2f**

^19^F NMR spectrum of compound **2f**

^1^H NMR spectrum of compound **5a**

^13^C NMR spectrum of compound **5a**

^1^H NMR spectrum of compound **5b**

^13^C NMR spectrum of compound **5b**

^1^H NMR spectrum of compound **5c**

^13^C NMR spectrum of compound **5c**

^1^H NMR spectrum of compound **5d**

^13^C NMR spectrum of compound **5d**

^1^H NMR spectrum of compound **5e**

^13^C NMR spectrum of compound **5e**

^1^H NMR spectrum of compound **5f**

^13^C NMR spectrum of compound **5f**

^1^H NMR spectrum of compound **5g**

^13^C NMR spectrum of compound **5g**

^19^F NMR spectrum of compound **5g**

^1^H NMR spectrum of compound **5h**

^13^C NMR spectrum of compound **5h**

^19^F NMR spectrum of compound **5h**

^1^H NMR spectrum of compound **5i**

^13^C NMR spectrum of compound **5i**

^19^F NMR spectrum of compound **5i**

^1^H NMR spectrum of compound **5j**

^13^C NMR spectrum of compound **5j**

^19^F NMR spectrum of compound **5j**

^1^H NMR spectrum of compound **5k**

^13^C NMR spectrum of compound **5k**

^19^F NMR spectrum of compound **5k**

^1^H NMR spectrum of compound **5l**

^13^C NMR spectrum of compound **5l**

^19^F NMR spectrum of compound **5l**

^1^H NMR spectrum of compound **6k**

^13^C NMR spectrum of compound **6k**
